# Supplementary material for: Ordovician opabiniid-like animals and the role of the proboscis in euarthropod head evolution
Source: Nat Commun. 2022 Nov 15;13:6969. doi: 10.1038/s41467-022-34204-w (PMC9666559; doi:10.1038/s41467-022-34204-w)
Supplement: Supplementary file 1 — Supplementary Information [file 41467_2022_34204_MOESM1_ESM.pdf]

Supplementary Information

Ordovician opabiniid-like animals and the role of the proboscis in euarthropod head evolution

Stephen Pates[1], Joseph P Botting[2, 3], Lucy A Muir[3] and Joanna M Wolfe[4]

[1] Department of Zoology, University of Cambridge, Downing Street, Cambridge, CB2 3EJ, UK. Corresponding author: sp587@cam.ac.uk

[2] Nanjing Institute of Geology and Palaeontology, Chinese Academy of Sciences, 39 East Beijing Road, Nanjing 210008, China.

[3] Department of Natural Sciences, Amgueddfa Cymru—National Museum Wales, Cathays Park, Cardiff CF10 3NP, UK.

[4] Museum of Comparative Zoology and Department of Organismic and Evolutionary Biology, Harvard University, 26 Oxford Street, Cambridge, MA 02138, USA

Supplementary Notes

Systematic Palaeontology

Mieridduryn bonniae nov. gen. et sp.

Further remarks.
The head region displays similarities with both opabiniids and radiodonts. Notably, an annulated proboscis was previously only known in opabiniids [1,2], while the presence of dorsal spines on the protocerebral appendage was previously only reported from radiodonts [3–5].

The presence of a dorsal sclerite in *Mieridduryn* distinguishes it from opabiniids. The dorsal position of the sclerite is comparable to radiodonts. Amplectobeluids and anomalocaridids display small subcircular dorsal sclerites, broadly comparable to *Mieridduryn*, however the outline of the sclerite in *Mieridduryn* is not clear enough to allow a detailed comparison [4,6]. Hurdiid radiodonts generally display much larger dorsal sclerites [7–9]. *Mieridduryn* lacks evidence for paired lateral sclerites, such as those known from amplectobeluids [6,10,11] and hurdiids [7]. As the dorsal sclerite of radiodonts is presumed homologous to the anterior sclerite of deuteropods [12], this morphological feature does not necessarily align *Mieridduryn* with radiodonts but instead with taxa which diverge later in the euarthropod stem lineage than opabiniids.

The remainder of the head region displays more similarities with taxa diverging earlier in the euarthropod stem-lineage, such as opabiniids and 'gilled lobopodians' *Pambdelurion* and *Kerygmachela*. Both *Mieridduryn* and *Opabinia* display a j-shaped gut that twists ventrally as it reaches the mouth (e.g. Ref [1] pl. XI, XIV, figs 55, 73). The presence of an oral cone composed of plates subequal in size in *Mieridduryn* is most comparable to the plates in the mouth apparatus of *Pambdelurion* [13], as it lacks the multiple sizes of plates diagnostic of radiodonts [14–16]. Some *Opabinia* specimens preserved as lateral compressions also show mouthparts with closely spaced parallel lines (e.g. Ref [1] pl. XI, fig. 55). Comparison with *Mieridduryn*, which displays an oral cone composed of plates subequal in size, suggests that the closely spaced parallel lines in the mouthparts of *Opabinia* could represent a laterally compressed oral cone. Similar laterally compressed oral cones have been reported from radiodonts, for example *Hurdia* (Ref [17] fig. 2D, E),.

The dorsolateral flaps and triangular lobopodous limbs of *Mieridduryn* are also very similar in outline and size to those described in *Opabinia* [18], as well as *Kerygmachela* and *Pambdelurion* [19,20]. *Mieridduryn* differs as it displays posterior-facing spines on its lobopodous limbs. Spinose lobopodous limbs are known in many lobopodians, including *Diania* (spines on multiple appendage margins [21]), and *Xenusion* (spines on posterior margin only [22]). While the subrectangular outline to the flaps bears a resemblance to *Opabinia,* the presence of internal strengthening rays that run parallel to the long axis of the flaps is well known in both the dorsal and ventral flaps of radiodonts [9]. In opabiniids (*Opabinia* and *Utaurora*) only a strengthened anterior margin to the swimming flaps has been reported [2].

Interpretation of the dark linear structures in the anterior flap as strengthening rays and setal structures is supported through comparison with opabiniid and radiodont material. Fine hair-like setal structures have been described in opabiniids *Opabinia* and *Utaurora* [2,18] and radiodonts including *Anomalocaris* [4]*.* Thus the hair-like structures protruding from beneath the posterior margin of the flaps are confidently identified as setal structures. The dark region beneath the anteriormost flap is interpreted as overlying setal structures and strengthening rays. Dark regions with fine hair-like details have been described in radiodont material as setal structures (in both *Anomalocaris canadensis* and *Cambroraster falcatus* [4,8]) while dark linear structures running parallel to the long

axis of the flap have been reported in numerous radiodonts, including *Buccaspinea cooperi* [23], where these features were quite faint, similar to *Mieridduryn*.

Although morphological comparisons support the interpretation of hair-like structures in *Mieridduryn* as setal blades, this new taxon is unique among stem group euarthropods in the arrangement of its setal blades. These appear to attach to the surface of the flap facing the body midline, as they are only visible protruding from underneath the flap in lateral view, or when the surface of the flap is split showing a cross section (**Figure 2b** in main text). In *Kerygmachela, Opabinia* and *Pambdelurion* setal blades attach to the dorsal surface of the flap [18,19,24], while in *Utaurora* they attach to the dorsal margin of the body and outer surface of the flap [2]. Radiodont setal blades also generally attach dorsally [4,7,9], with the exception of *Cambroraster,* where they have been suggested to attach to the ventral surface of the body [8].

Castle Bank euarthropod A (NMW.2021.3G.8)

Further remarks.
The head region displays similarities with *Mieridduryn*, opabiniids and radiodonts. In particular, the presence of an internal canal within a proboscis has previously been recognised in *Opabinia* and possibly *Utaurora* [1,2], while the presence of dorsal spines on a proboscis is shared with *Mieridduryn*. NMW.2021.3G.8 can be distinguished from *Mieridduryn* as its spines have a broader base and are shorter than those of *Mieridduryn,* although this difference could be ontogenetic rather than diagnostic of a new species.

The presence of lateral sclerites in the head region is unknown in any opabiniid or *Mieridduryn*. Lateral sclerites are known in at least two radiodont families (amplectobeluids and hurdiids) where they are associated with a central dorsal sclerite to form a tripartite carapace complex [6,7]. The presence of a dorsal sclerite cannot be confirmed in NMW.2021.3G.8, and its carapace is not as large as that of hurdiids, where it can reach up to 50% of the body length [7–9]. The coverage of the lateral margins of the head and proximal part of the proboscis is more comparable to what is observed in amplectobeluids, where the carapace elements cover the proximal parts of the appendages [6,10]. The lateral elements of NMW.2021.3G.8 differ from both radiodont families in its shape and the presence of anterior facing marginal spines. No radiodont lateral element displays a spinose anterior margin, however some hurdiid central elements do bear spines (e.g. *Cambroraster, Pahvantia* [8,23]). *Pambdelurion whittingtoni* also displays anterior cephalic spines [13], however in the Sirius Passet taxon these spines are not associated with any carapace element. Paired spinose carapaces are also known in some upper stem group bivalved euarthropods, such as *Isoxys* and especially *Tuzoia* [25,26]. It is uncertain whether the two carapace elements are joined in NMW.2021.3G.8, or if there are additional elements between them, and not enough detailed morphological similarities can be drawn between the lateral carapace of NMW.2021.3G.8 and radiodonts to confirm that they are homologous. Thus, while it is possible that the paired lateral carapace elements of NMW.2021.3G.8 could provide a link between the carapaces of lower stem group euarthropods and bivalved forms - indeed previous workers have suggested that they might be homologous structures [27] - a viable alternative is that carapaces evolved independently in radiodonts, NMW.2021.3G.8 and bivalved euarthropods.

The status of the subrectangular elements (**re** in main text **Figures 6, 7a**) situated posterior to the lateral carapace elements is also problematic. Other sclerotized features in closely related euarthropods tend to be ventrally located. These include gnathobase like structures of amplectobeluid radiodonts, which are situated posterior to the mouth [6,15] and hourglass shaped plates in *Parapeytoia* which are considered sternites[28]. Thus a dorsal position for the subrectangular

lightly sclerotized features in NMW.2021.3G.8 precludes homology with these other features. An interpretation of the subrectangular plates as tergites is also unfavourable, as they do not have a regular shape or outline, and do not even cover the whole dorsal surface laterally. Thus at this time these posterior elements appear to have no comparable feature in lower stem group euarthropods nor deuteropods, though they may plausibly represent a broken posterior margin of the larger sclerites (**ls** in main text **Figures 6, 7a, Supplementary Figure 5**).

The remainder of the body is most similar to *Mieridduryn* and opabiniids, although additional similarities with some radiodonts can also be noted. The narrow body profile of NMW.2021.3G.8 is comparable to both opabiniids and the hurdiid radiodonts *Aegirocassis* and *Hurdia,* however dorsal intersegmental furrows are only known in *Mieridduryn* and opabiniids [2,9,17]. The subrectangular outline of the flaps is shared with *Opabinia* and *Mieridduryn*, and the number of caudal blades is also shared with the former (caudal blades are not visible on the latter). However, the shape of the caudal blades is more similar to those of the opabiniid *Utaurora* [2,18] and some radiodonts (e.g. *Anomalocaris, Houcaris, Lyrarapax* [4,10,29]). Further similarities with opabiniids come from the spinose nature of the caudal blades. *Opabinia* and *Utaurora* both have spinose caudal blades [2]. No spines have been reported from any caudal structures in radiodonts. The absence of rami in NMW.2021.3G.8 represents a difference from opabiniids, as paired caudal rami are known in both *Opabinia* and *Utaurora*[2], as well as many radiodonts [e.g. 29].

Supplementary Discussion

Sensitivity of phylogenetic results

Comparison of different models and different numbers of Castle Bank terminals.

Phylogenetic methods model evolution in different ways, and the relative merits and weaknesses of Maximum Parsimony (MP) and Bayesian Inference (BI) analyses for addressing phylogenetic questions have been analysed [30,31]. Following Ref [2] we interrogated our results with three different phylogenetic approaches, visualised the results in multidimensional treespace, and calculated the number of retrieved topologies in support of different hypotheses. Furthermore, as it remains uncertain how many taxa the two Castle Bank specimens represent, we compared the results between analyses considering one and two Castle Bank terminals.

The three approaches used were: MP with concavity constant k = 3 (further details following Ref [32]), and the maximum information and minimum assumptions Bayesian Inference strategies of Ref [33]. The maximum information strategy aims to maximise the information content of the morphology, but can potentially misinterpret phylogenetic noise as true signal. The minimum assumptions hyperpriors instead aim to minimise *a priori* assumptions, with the potential cost of decreased phylogenetic certainty. The two strategies also differ in the Dirichlet hyper-priors used[34]. The maximum information strategy imposes a fairly constrained model, allowing only symmetrical rates of character change (reversal is as likely as forward transition), while the minimum assumption strategy allows asymmetric rates of character change.

1. *Results and Discussion.*

When the two Castle Bank specimens were considered as distinct species, all three approaches resolved the two Castle Bank taxa crownwards of *Opabinia* and *Utaurora*, sister to radiodonts + deuteropods. As expected, the support values at most nodes in the BI minimum assumptions analysis were lower than for the maximum information analysis (**Supplementary Figure 6**). When the two Castle Bank specimens were considered one terminal, the maximum information and MP analyses resolved the Castle Bank terminal in a crownwards position from *Opabinia* and *Utaurora*, i.e. sister lineage to radiodonts + deuteropods (**Supplementary Figure 7a, c**). The minimum assumptions analysis instead recovered a polytomy of *Opabinia, Utaurora* and the Castle Bank terminal (**Supplementary Figure 7b**).

Breakdown of the phylogenetic support in favour of a monophyletic group of proboscis-bearing euarthropods (Castle Bank + *Opabinia* + *Utaurora*) and the alternative in favour of a paraphyletic grade (Castle Bank in a crownwards position relative to *Opabinia* and *Utaurora*) is provided in **Supplementary Tables 1, 2.** Trees recovering the Castle Bank specimens in a monophyletic group with *Opabinia* and *Utaurora* only, or with both opabiniids and radiodonts, were counted as supporting a monophyletic group of proboscis-bearing animals. Trees recovering the Castle Bank specimens in a monophyletic group with deuteropods, or with deuteropods and radiodonts (but not including *Opabinia* and *Utaurora*), were counted as supporting the paraphyletic grade of proboscis-bearing animals hypothesis. An additional few trees which recovered the Castle Bank specimens in a monophyletic group with radiodonts, but not including *Opabinia* or *Utaurora,* were also counted in support of this hypothesis. These data reveal that nodal support for a paraphyletic grade of proboscis-bearing euarthropods in the analyses considering the Castle Bank specimens to represent one terminal is lower, while the alternative (a monophyletic group of proboscis-bearing euarthropods) is increased when Castle Bank specimens represent two terminals (**Supplementary Tables 1, 2**). This is reflected in the treespace visualisations (**Supplementary Figures**

8, 9). When two Castle Bank terminals are considered, the treespace lacks a clear structure, and the area occupied by trees supporting monophyletic or paraphyletic proboscis-bearing euarthropods nearly completely overlap (**Supplementary Figure 8b**). This indicates that variation in the position of the Castle Bank specimens is not the major cause of difference between topologies, which is also supported by the relatively strong support (in terms of number of retrieved trees) for one hypothesis over the other. The treespace visualisations of the analyses considering the Castle Bank specimens as a single terminal display two overlapping islands of trees, one supporting the monophyletic proboscis group, and the other a paraphyletic proboscis grade (**Supplementary Figure 9b**). The support for one hypothesis over the other does not appear to correlate with either maximum information or minimum assumptions tree islands. The MP trees fall within the paraphyletic proboscis data ellipse (**Supplementary Figure 9a**). Importantly, regardless of phylogenetic model or number of Castle Bank terminals considered, support for a paraphyletic grade of proboscis-bearing euarthropods outweighs support for the alternative - that these taxa instead form a monophyletic group.

Further differences in the consensus trees can be observed, which do not directly impact the conclusions of this study. Both MP analyses recovered monophyletic Radiodonta, whereas among the BI analyses, only the minimum assumptions analysis with two Castle Bank terminals recovered this result. The others instead recovered monophyletic Hurdiidae, and a monophyletic group of Amplectobeluidae + Anomalocarididae, in a polytomy with Deuteropoda. The MP analyses also both recovered monophyletic opabiniids (*Opabinia* + *Utaurora*), however the BI analyses instead recovered a polytomy of opabiniids. Interrogation of the number of trees in support of monophyletic opabiniids revealed that, although support for this node was <50% for the BI analyses, it is still better supported than the alternatives (*Utaurora* crownwards of *Opabinia*, or *Opabinia* crownwards of *Utaurora*). Support for this node has likely decreased compared to the previous study [2] as characters previously only found in opabiniids (proboscis, spinose caudal blades, dorsal furrows) are now also shared with the Castle Bank specimens.

Assumption: Including additional deuteropod taxa will not change topology of lower stem group below radiodonts + deuteropods

This manuscript seeks to understand the phylogenetic position of the new Castle Bank specimens, and more broadly the topology of the lower stem group of Euarthropoda. While numerous different hypotheses have been put forward for the group that should be considered sister to all other deuteropods, the inclusion or exclusion of particular deuteropod taxa was assumed not to change the relationships of taxa in the lower stem group of euarthropods, specifically diverging prior to the split between radiodonts and deuteropods.

In the analyses presented in the main text, fossil deuteropods are represented by artiopodans, megacheirans and fuxianhuiids. Other workers have suggested that either *Kylinxia zhangi* or *Parapeytoia yunnanensis* may be sister groups to all other deuteropods [28,35]. Support for *Kylinxia zhangi* as sister group to all other deuteropods comes from the apparent similarity in the morphology of its frontalmost appendages to the frontal appendages of radiodonts. Zeng et al. [35] consider that the protocerebral appendages of radiodonts may have migrated to the deutocerebrum in *Kylinxia*. Budd [28] instead sees *Parapeytoia* as a crucial taxon. Budd interprets the frontalmost appendages of *Parapeytoia* as protocerebral, and reinterprets the neurological data in support of a deutocerebral origin for megacheiran appendages [36,37] and labrum [38] to argue that megacheirans also possessed protocerebral appendages.

To ensure that inclusion of these taxa did not impact on the recovered topology of the lower stem group, specifically the relationships of opabiniids and the Castle Bank species, a series of sensitivity analyses were run using the maximum information and minimum assumptions BI strategies. These additional analyses included *Kylinxia* and *Parapeytoia*, in addition to the taxa in the analyses of the main text. When coding the segmental affinities of the raptorial appendages in these two extra taxa, we treated *Kylinxia* appendages as deutocerebral (following Ref [35]), and *Parapeytoia* as protocerebral (following Ref [28]) in one set of analyses, and deutocerebral (e.g. Ref [9]) in a second.

Results and Discussion.

The BI maximum information analysis resolved the same topology of lower stem group euarthropods, with a paraphyletic grade of proboscis-bearing taxa (*Opabinia, Utaurora* and the two Castle Bank species) forming the sister group to radiodonts and deuteropods. *Kylinxia* was always retrieved well within Deuteropoda, and *Parapeytoia* was always resolved as sister group to deuteropods (**Supplementary Figures 10, 11**). For the maximum information strategy considering *Parapeytoia* as possessing protocerebral appendages, radiodonts were resolved as paraphyletic, with 53% of trees placing hurdiids closer to deuteropods than to other radiodonts (**Supplementary Figure 10a**). The BI minimum assumptions analysis where *Parapeytoia* appendages were considered deutocerebral recovered monophyletic Radiodonta (**Supplementary Figure 11b**). The other two analyses resolved a polytomy of radiodonts, with one group comprised of hurdiids, and the other amplectobeluids + anomalocaridids (**Supplementary Figures 10b, 11a**).

When all four sensitivity analyses are considered together, it appears that the incorporation of additional taxa that are putatively transitional between radiodonts and deuteropods does not alter the paraphyletic grade of proboscis-bearing euarthropods within the euarthropod stem lineage. This holds even when *Parapeytoia* is considered to possess protocerebral frontal appendages. Comparison of results of analyses considering *Parapeytoia* frontal appendages as protocerebral and deutocerebral respectively indicate that the phylogenetic position of *Parapeytoia* as a sister group of deuteropods is not driven by the segmental affinity of the frontal appendages, as *Parapeytoia* occupies the same position in both sets of analyses (**Supplementary Figures 10, 11**). It is most likely that other morphological features of *Parapeytoia,* such as its unarthrodized body and oral cone, cause it to be resolved stemwards from other deuteropods, while the presence of deuteropod characters such as a biramous limb cause it to fall closer to deuteropods than to radiodonts and other members of the lower stem group.

Considering *Parapeytoia* frontal appendages as protocerebral did impact on the topology of radiodonts, as one analysis recovered weak support for radiodont paraphyly. Thus, if *Parapeytoia* appendages are considered protocerebral, and the segmental affinity of euarthropod appendages of Budd [28] is followed, then our results suggest a more convoluted evolution of the protocerebral appendages in total-group euarthropods. These assumptions give two possible interpretations for the evolution of protocerebral appendages in euarthropods. Firstly, the protocerebral appendage may have fused in the common ancestor of *Opabinia* and deuteropods, before becoming unfused and separating into paired arthropodized appendages in the common ancestor of radiodonts and deuteropods, before fusing and reducing into the labrum in deuteropods. The alternative is that the protocerebral appendages fused independently in opabiniids, the Castle Bank taxa, and deuteropods.

Budd [28] draws comparisons between *Parapeytoia* and amplectobeluid radiodont features, such as the reduced 'neck' region and gnathobase-like structures. Thus a paraphyletic grade resolving hurdiids closer to *Parapeytoia* and deuteropods than to other radiodonts is problematic for

drawing homology between features that are present in amplectobeluids but absent in hurdiids (e.g. gnathobase-like-structures) and morphologically similar features in *Parapeytoia* (e.g. gnathobasic protopodite). The plate-like, unpaired, endites present on the appendages of both hurdiid radiotonts and *Parapeytoia* does support a closer association, than with amplectobeluids and anomalocaridids, if, as in these analyses, the appendages are considered homologous.

If *Parapeytoia* appendages are not considered homologous to radiodont frontal appendages, but instead are considered to be innervated by the deutocerebrum, the results are directly comparable to analyses presented in the main text (**Figure 10, Supplementary Figures 6, 7**). These results suggest a less convoluted pathway for the evolution of protocerebral appendages. This scenario indicates that the protocerebral appendages of radiodonts are unique in being paired and arthropodized, while those of upper stem group euarthropods and proboscis-bearing lower stem euarthropods are fused. As indicated by its position closer to deuteropods than to radiodonts in our analyses, *Parapeytoia yunnanensis* would still be considered a crucial taxon for understanding the sequence of evolution of characters within the euarthropod stem lineage, even with deutocerebral raptorial appendages. Unlike megacheirans, *P. yunnanensis* possesses an oral cone, and its trunk is also unarthrodized with the exception of gnathobasic limbs and hourglass shaped 'sternites' [28,39]. Thus, with the exception of the morphology of its frontalmost appendages, it is quite different from megacheirans.

Importantly, regardless of the inclusion/exclusion of *Kylinxia* and *Parapeytoia,* and the coding of *Parapeytoia* appendages as innervated by the protocerebrum or deutocerebrum, a paraphyletic grade of proboscis-bearing euarthropods with a posterior facing mouth is consistently resolved within the lower stem group of Euarthropoda.

Radiodonts are unique in possessing arthropodized protocerebral appendages

The similarity in the morphology of the frontal appendages of radiodonts and other stem euarthropods, such as *Kylinxia,* megacheirans, *Parapeytoia* and isoxyids, has been used to argue for homology in the frontalmost appendage of these groups [28,35,40,41] either as protocerebral [28] or deutocerebral [41]. Support for these hypotheses requires either reinterpreting or discarding palaeoneurological data and evidence for a labrum in megacheirans [36–38,42], or a segmental transformation of the appendages from protocerebral to deutocerebral between radiodonts and deuteropods [35]. However, other morphological features of *Kylinxia,* megacheirans, and *Parapeytoia* are not necessarily congruent with considering the frontalmost appendages and homologous to radiodonts.

Of upper stem group euarthropods, *Kylinxia* displays the most strikingly similar appendages to those of radiodonts, specifically amplectobeluids and anomalocaridids [35]. However, the many differences in the bodies of *Kylinxia* and radiodonts, such as a toughened and fully segmented dorsal exoskeleton and biramous limbs in *Kylinxia*, undermines support for *Kylinxia* as sister group to all other deuteropods, especially because taxa with biramous limbs and unarthrodized bodies, such as isoxyids and *Parapeytoia,* are known in the fossil record [28,43].

Parapeytoia has also been suggested to occupy a pivotal position between radiodonts and deuteropods [28], indeed our additional phylogenetic analyses support this position (**Supplementary Figures 10, 11**). *Parapeytoia* is known from slightly disarticulated material. The head region bears paired arthropodized appendages anterior to an oral cone, while the trunk is not arthrodized, but does possess hourglass shaped sternites and biramous limbs with robust gnathobases [39,44]. The

appendages of *Parapeytoia* are more similar to those of megacheirans than those of radiodonts, but the unarthrodized body of *Parapeytoia,* the structure of its gnathobases, and the presence of an oral cone and dorsolateral body flaps have all prompted comparisons to radiodonts [39,44]. However further interrogation of the radiodont-like characters does not support a closer association with Radiodonta, but rather lower stem group euarthropods in general. A key character known only in radiodonts - the presence of multiple sizes of plate in the oral cone - cannot be confirmed from the published images of *Parapeytoia* (*contra* Ref [28]). An oral cone (without plates of different sizes) is present within the lower stem group more generally, for example in *Mieridduryn* and *Pambdelurion* (this study; Ref [13]). Comparisons have also been drawn between the gnathobases of *Parapeytoia* and the gnathobase-like structures of amplectobeluids [28]. However, as noted in the original description of gnathobase-like structures in *Amplectobelua,* these structures could represent the proximal region of an arthropodized appendage (thus comparable to *Parapeytoia* gnathobases), but they could also represent the distal part of an appendage [6]. Other shared features between radiodonts and *Parapeytoia,* such as dorsolateral flaps with strengthening rays, are also shared with *Mieridduryn*, while an unarthrodized body is present in all lower stem group euarthropods and isoxyids [43,45] as well as putative 'transitional taxon' between lower and upper stem groups, *Erratus sperare* [46]. Thus, while the combination of characters in *Parapeytoia* are intriguing and this species likely is important for understanding the transition between the lower and upper stem groups of Euarthropoda, the currently known material does not provide strong support for an especially close association with Radiodonta. Furthermore, segmental homology of *Parapeytoia* and radiodont frontalmost appendages is not necessary for *Parapeytoia* to occupy this pivotal phylogenetic position (**Supplementary Figures 10, 11**), though our matrices do not include *E. sperare*.

A raptorial head appendage has evolved numerous times within Panarthropoda [47] and more specifically within Euarthropoda, for example in the bivalved euarthropod *Clypecaris* [48] and the putative artiopodan *Kodymirus* [49]. The presence of dorsal spines on the proboscises of the Castle Bank specimens adds further support for considering the anteriormost appendages of *Kylinxia* and *Parapeytoia,* and of megacheirans more broadly, as segmentally and evolutionarily distinct from radiodont frontal appendages. Radiodont appendages are united by the presence of at least one podomere bearing a dorsal spine, though often dorsal spines have no clear function. The presence of dorsal spines on the protocerebral appendages of the Castle Bank specimens – which do not form a clade with radiodonts – and the presence of at least one dorsal spine on the appendages of all known radiodonts, suggests that this feature may have been ancestral within Radiodonta (assuming that they did not evolve convergently, which is also possible). The absence of dorsal spines in *Kylinxia* and *Parapeytoia* may be a phylogenetically informative signal indicating a segmental difference between the frontal appendages of these two taxa and radiodonts.

Dorsal spines and arthropodization of radiodont protocerebral appendages

The presence of dorsal spines in the Castle Bank specimens may also provide insight on the origin of the arthropodized protocerebral appendages in radiodonts. These spines could have strengthened the dorsal margin, and demarcated the proboscis into distinct regions. Jointed appendages may have developed via the co-option of a coordinate system of regularly spaced features on an unjointed limb [50]. Thus, arthropodized radiodont appendages may have resulted from the subsequent strengthening and sclerotization of these regions separated by dorsal spines. Support for this hypothesis comes from the location of the dorsal spines in radiodont appendages. These dorsal spines attach at the distalmost point of the podomere (e.g. *Amplectobelua, Anomalocaris, Houcaris, Paranomalocaris* [4,51–53]), and are thus are associated with pivot points at the apex of the triangular membranes. Furthermore, proximal dorsal spines in radiodont appendages tend to be reduced in

size, and commonly lack any apparent function. Often they are visible only as circular bases (e.g. *Houcaris, Paranomalocaris* [52,53]), or absent on most podomeres altogether (e.g. *Anomalocaris, Amplectobelua* [3,4,51]). It is only distally that dorsal spines become prominent, often hypertrophied and recurved, and even then only in some taxa (e.g. *Amplectobelua*; *Anomalocaris* [3,4,51]). This suggests that distal dorsal spines in some radiodonts were co-opted for prey capture, while others became vestigial, once sclerotisation of podomeres made their original role (strengthening the dorsal margin) obsolete.

Supplementary Tables

	All trees	BI only	MP only
Paraphyletic proboscis	9148 (76%)	9139	9
Monophyletic proboscis	1643 (14%)	1643	0
Other	1235 (10%)	1235	0
Castle Bank taxa form monophyletic group with			
Radiodonta + Deuteropoda	8258 (69%)	8249	9
Opabiniidae (*Opabinia* and *Utaurora*)	1543 (13%)	1543	0
Deuteropoda	827 (7%)	827	0
Radiodonta	599 (5%)	599	0
Opabiniidae + Radiodonta ('Dinocarida')	115 (1%)	115	0
Other	1235 (10%)	1235	0

Supplementary Table 1. Breakdown of phylogenetic support for affinities of Castle Bank taxa, when treated as two species. Note that some trees support more than one group in subsection 'Castle Bank taxa form monophyletic group with' (because these may be nested clades), and so this column does not add up to 12017. Abbreviations: BI = Bayesian Inference; MP = Maximum Parsimony.

	All trees	BI only	MP only
Paraphyletic proboscis	7030 (59%)	7030	9
Monophyletic proboscis	3908 (33%)	3908	0
Other	1079 (9%)	1079	0
Castle Bank specimens form monophyletic group with			
Radiodonta + Deuteropoda	6228 (52%)	6219	9
Opabiniidae (*Opabinia* and *Utaurora*)	3837 (32%)	3837	0
Deuteropoda	780 (6%)	780	
Radiodonta	539 (4%)	539	0
Opabiniidae + Radiodonta ('Dinocarida')	90 (<1%)	90	0
Other	1079 (9%)	1079	0

Supplementary Table 2. Breakdown of phylogenetic support for affinities of Castle Bank specimens, when treated as a single species. Note that some trees support more than one group in subsection 'Castle Bank specimens form monophyletic group with' (because these may be nested clades), and so this column does not add up to 12017. Abbreviations: BI = Bayesian Inference; MP = Maximum Parsimony.

Castle Bank specimens form monophyletic group with	Carapace convergent	Dorsal spines convergent	Strengthening rays convergent	All convergent	No convergences
Opabiniidae (*Opabinia* and *Utaurora*)	6113 (82%)	3732 (62%)	2734 (46%)	5026 (84%)	1950 (32%)
Radiodonta + Deuteropoda	319 (4%)	1378 (23%)	2214 (37%)	153 (3%)	3298 (55%)
Deuteropoda	179 (2%)	294 (5%)	315 (5%)	142 (2%)	275 (5%)
Radiodonta	13 (<1%)	86 (1%)	146 (2%)	5 (<1%)	257 (4%)
Opabiniidae + Radiodonta ('Dinocarida')	48 (<1%)	40 (<1%)	43 (<1%)	20 (<1%)	49 (<1%)
Other	854 (11%)	636 (11%)	719 (12%)	680 (11%)	434 (7%)
Total number of trees	7484	6004	6004	6004	6004
Implication:	Monophyletic proboscis	Monophyletic proboscis	Monophyletic proboscis	Monophyletic proboscis	Paraphyletic proboscis

Supplementary Table 3. Breakdown of phylogenetic support for affinities of Castle Bank specimens, when treated as a single species, comparing different assumptions about characters being convergent/homologous between Castle Bank specimens and radiodonts. All analyses run under the 'maximum information' Bayesian Inference strategy, using a single Castle Bank terminal. Note that some trees support more than one group (because these may be nested clades), and so this column does not add up to the number in the total number of trees row.

Supplementary Figures

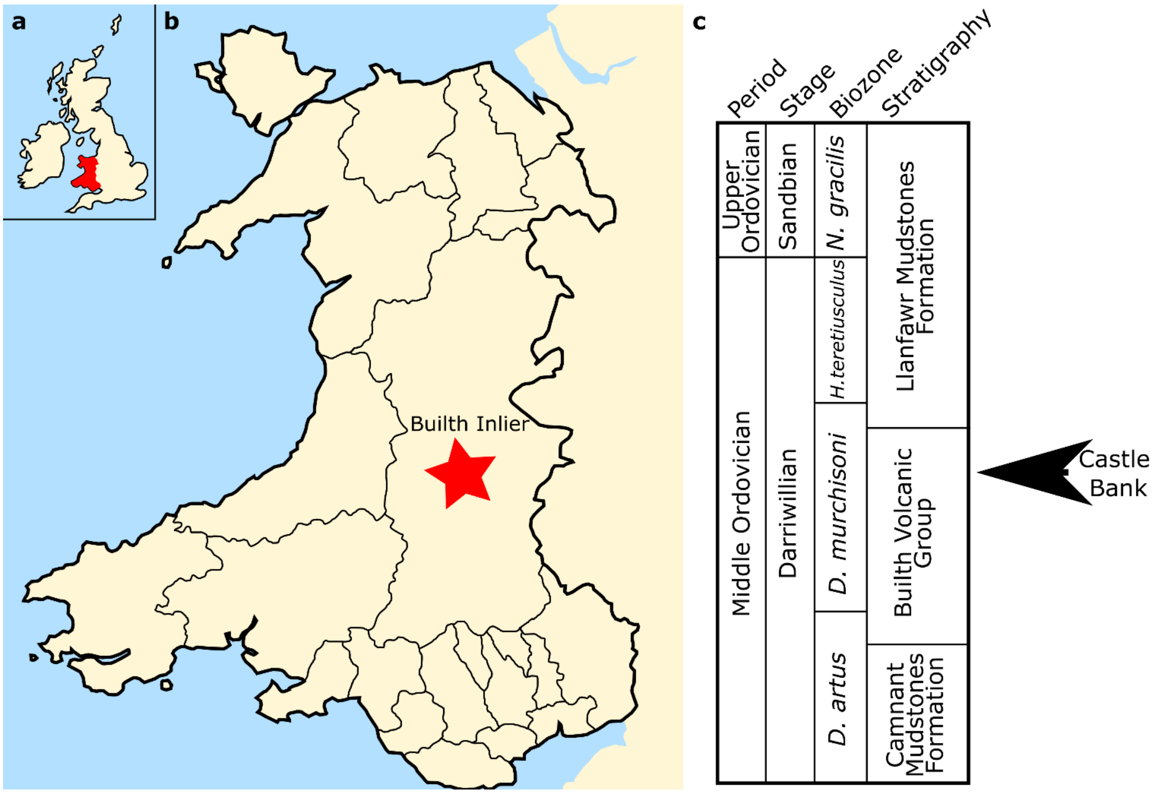

Supplementary Figure 1. Geographic and stratigraphic location of the Castle Bank Konservat-Lagerstätte. (a,b) Location of the Builth Inlier (Wales, UK). (c) Simplified stratigraphy of the northern part of the Builth Inlier sequence, with the approximate stratigraphic position of Castle Bank marked. Abbreviations: *D.*, *Didymograptus*; *H. Hustedograptus*; *N.*, *Nemagraptus*.

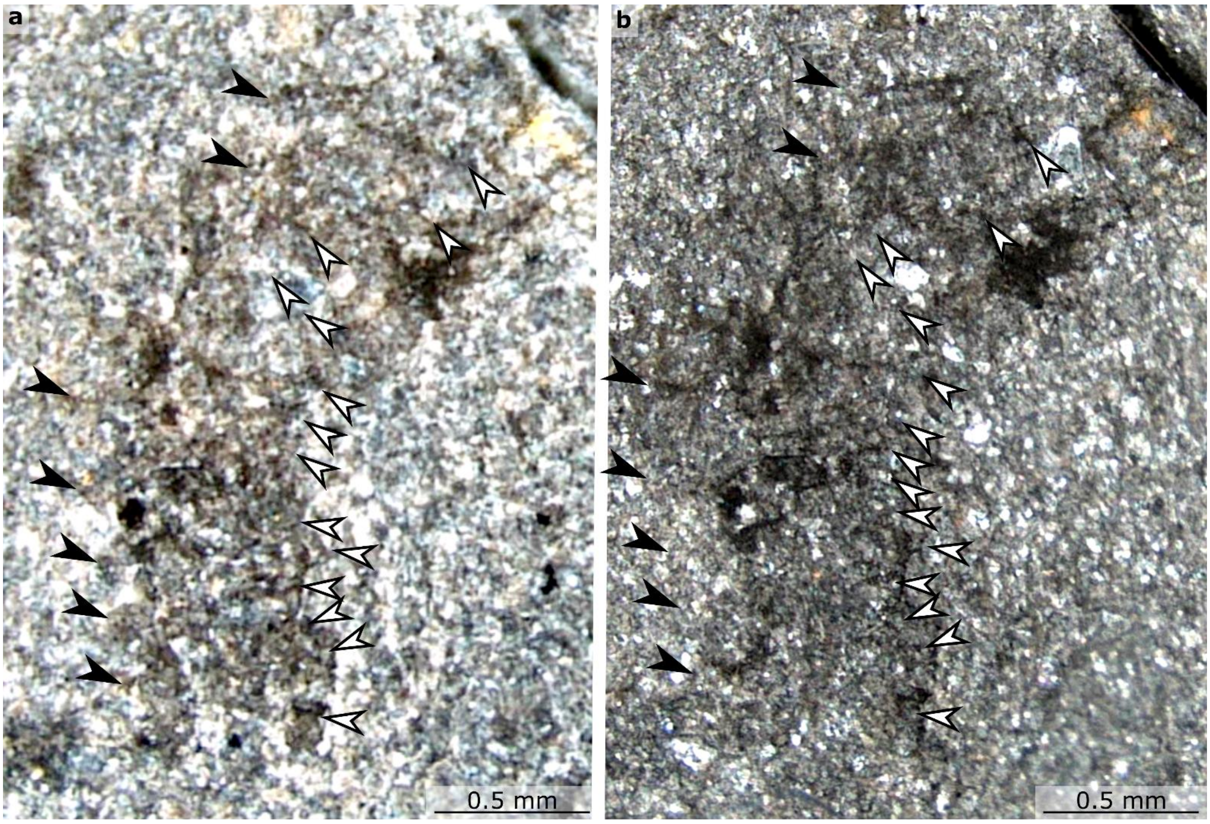

Supplementary Figure 2. *Mieridduryn bonniae* nov. gen. et sp. (NMW.2021.3G.7), details of the proboscis under different lighting conditions. Note how orientation of annulations and spines changes with curvature of the proboscis. (a) S8 microscope, high angle cross-polarized light with contrast increased. (b) M125 microscope, high angle light, contrast increased. White arrows indicate annulations, black arrows spines.

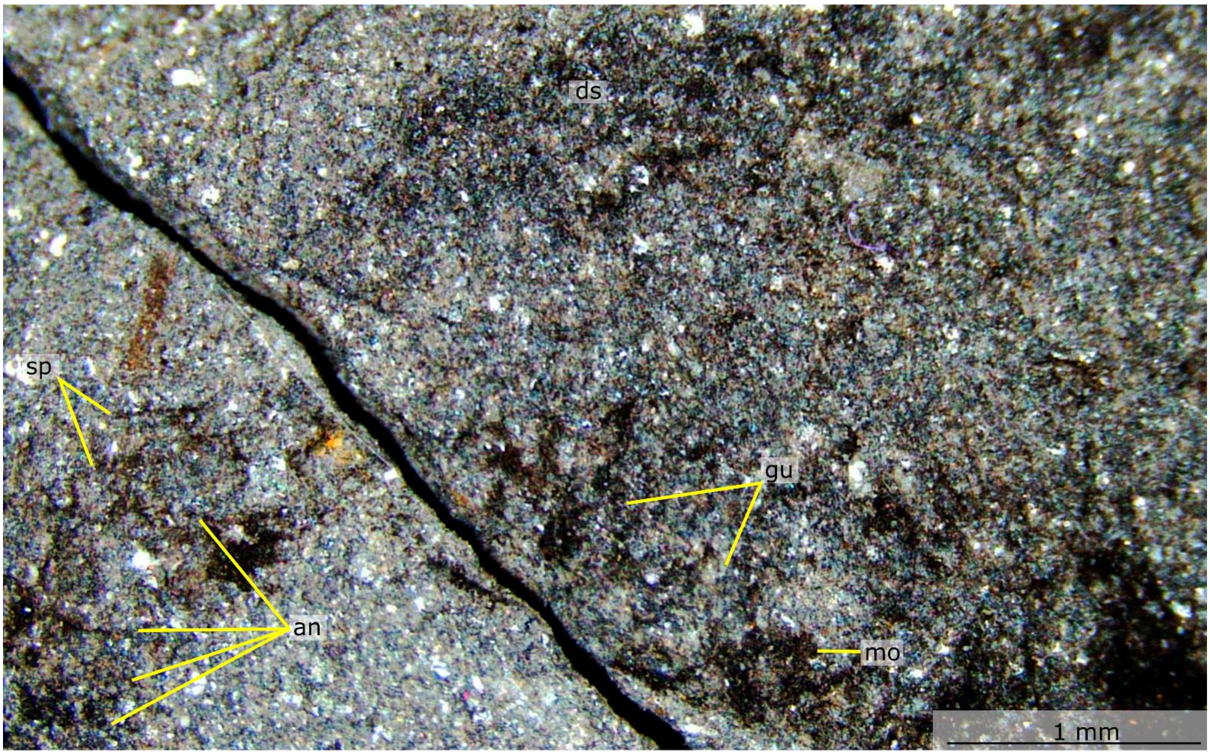

Supplementary Figure 3. *Mieridduryn bonniae* nov. gen. et sp. (NMW.2021.3G.7). Detailed view of the anterior sclerite, gut, and location of the oral cone. Abbreviations: an, annulation; gu, gut trace; mo, mouth and oral cone; sp, spine.

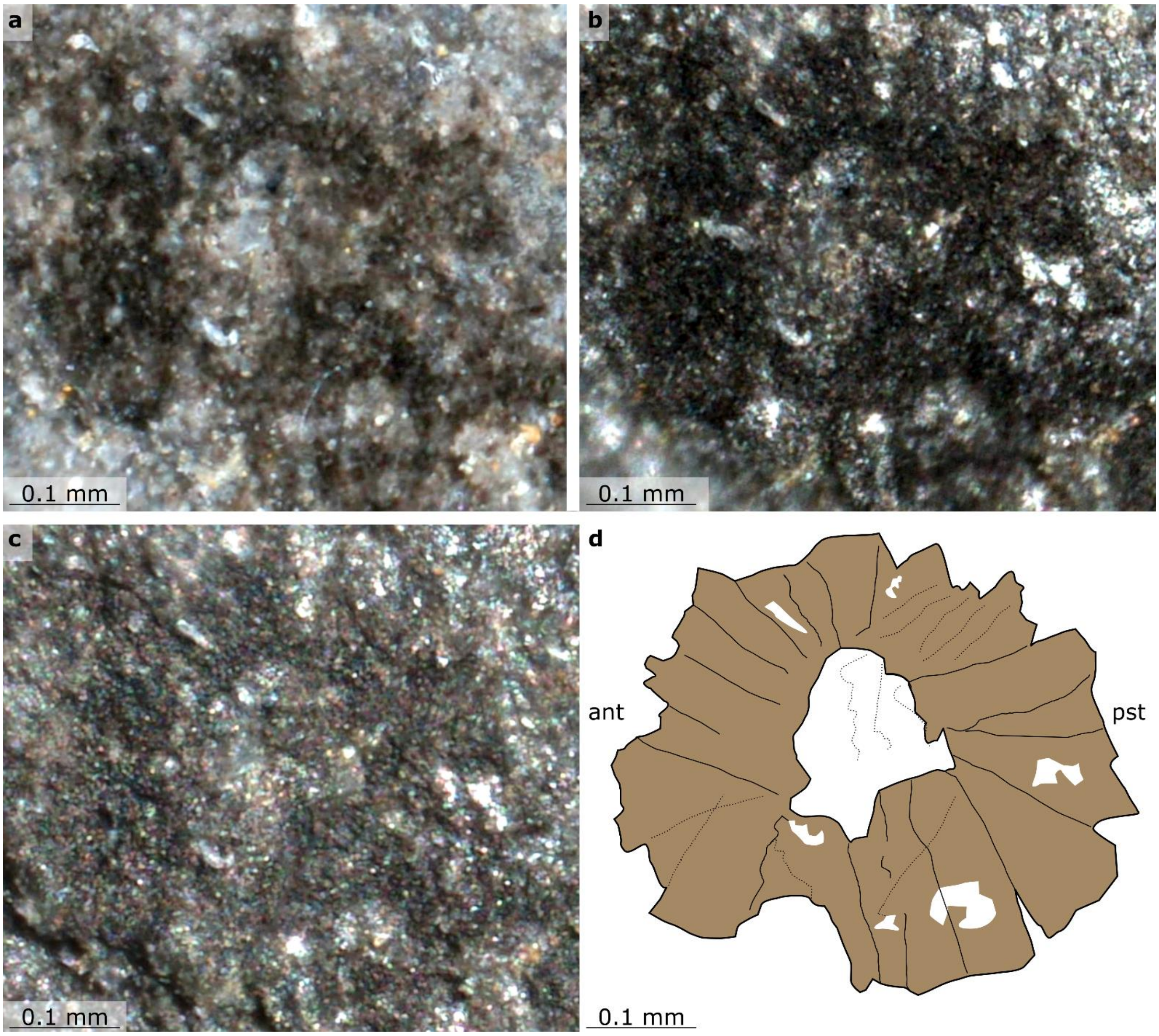

Supplementary Figure 4. *Mieridduryn bonniae* nov. gen. et sp. (NMW.2021.3G.7).(a–c) Details of the oral cone under different lighting conditions. (a) S8 microscope, high angle light. (b) M125 microscope, high angle light, contrast increased. (c) S8 microscope, low angle light. (d) Composite interpretative drawing. Abbreviations: ant, anterior; pst, posterior.

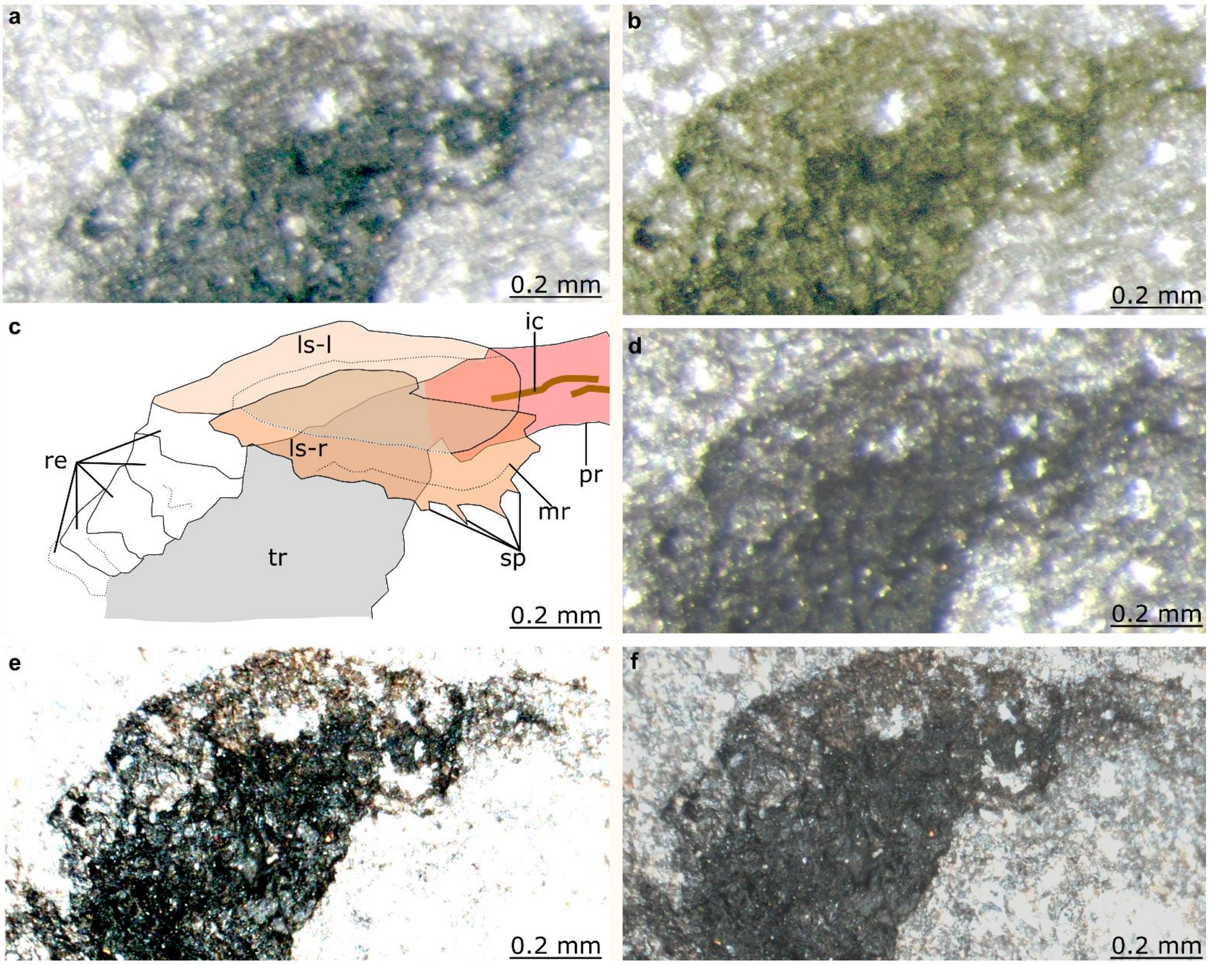

Supplementary Figure 5. Head region of Castle Bank euarthropod A (NMW.2021.3G.8), showing the proximal region of the proboscis and lateral sclerites in more detail, under different microscopes and lighting regimes, and interpretative drawing (c). (a) M125 microscope, high angle light. (b) M125 microscope, high angle light, yellow channel increased. (c) interpretative drawing). (d) M125 microscope, high angle light. (e) S8 microscope, high angle light, contrast increased. (f) S8 microscope, high angle light. Abbreviations: ic, internal canal of proboscis; ls-l, left lateral sclerite; ls-r, right lateral sclerite; mr, marginal rim to lateral sclerite; pr, proboscis; re, subrectangular elements posterior to lateral sclerites; sp, spine; tr, body trunk.

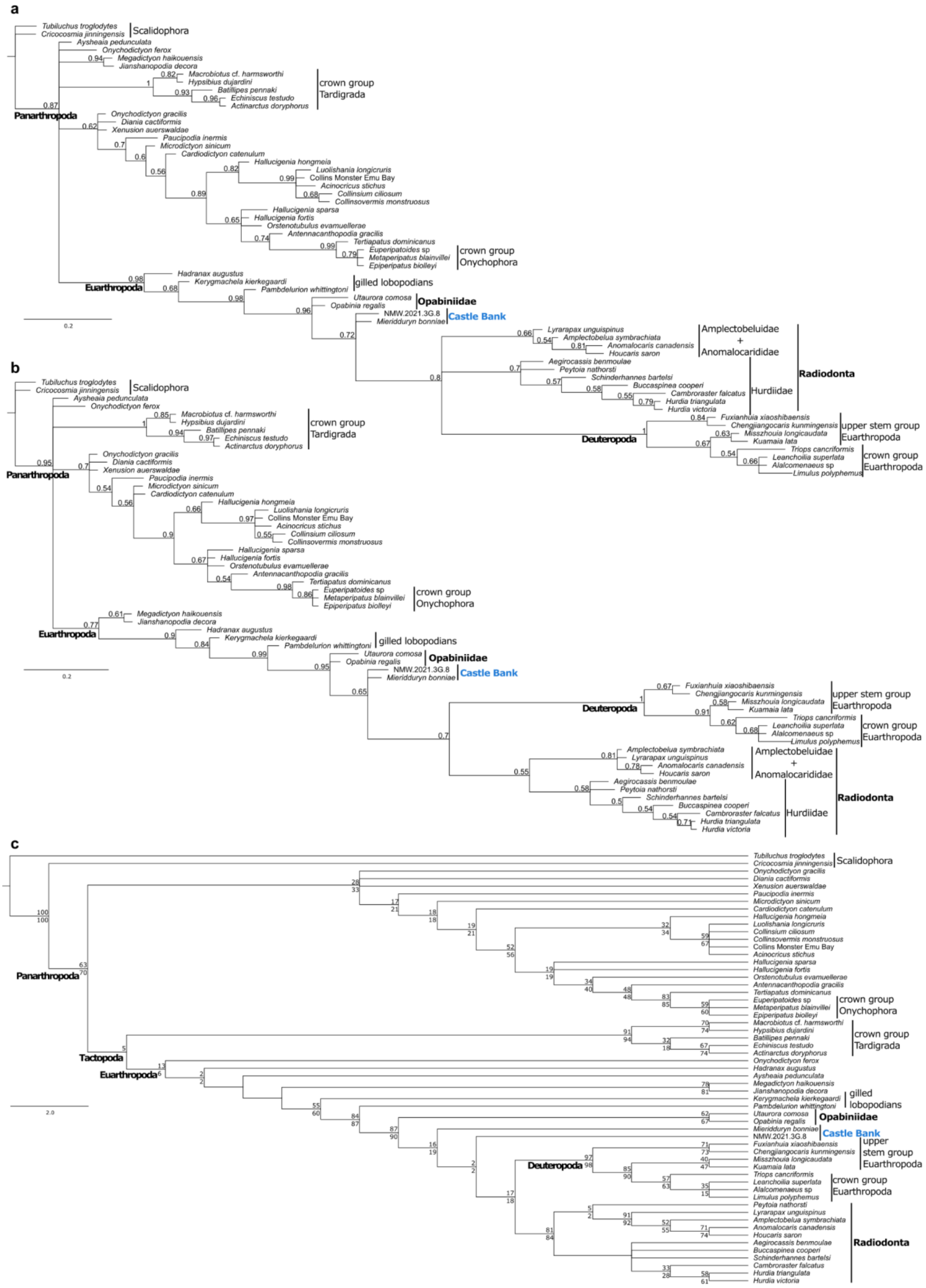
a
Tubiluchus troglodytes
Cricocosmia jinningensis
Scalidophora
Aysheaia pedunculata
Onychodictyon ferox
Megadictyon haikouensis
Jianshanopodia decora
Macrobiotus cf. harmsworthi
Hypsibius dujardini
Batillipes pennaki
Echiniscus testudo
Actinarctus doryphorus
crown group Tardigrada
Panarthropoda
Onychodictyon gracilis
Diania cactiformis
Xenusion auerswaldae
Paucipodia inermis
Microdictyon sinicum
Cardiodictyon catenulum
Hallucigenia hongmeia
Luolishania longicruris
Collins Monster Emu Bay
Acinocricus stichus
Collinsium ciliosum
Collinsovermis monstruosus
Hallucigenia sparsa
Hallucigenia fortis
Orstenotubulus evamuellerae
Antennacanthopodia gracilis
Tertiapatus dominicanus
Euperipatoides sp
Metaperipatus blainvillei
Epiperipatus biolleyi
crown group Onychophora
Euarthropoda
Hadranax augustus
Kerygmachela kierkegaardi
Pambdelurion whittingtoni
gilled lobopodians
Utaurora comosa
Opabinia regalis
Opabiniidae
NMW.2021.3G.8
Mieridduryn bonniae
Castle Bank
Lyrarapax unguispinus
Amplectobelua symbrachiata
Anomalocaris canadensis
Houcaris saron
Amplectobeluidae + Anomalocarididae
Aegirocassis benmoulae
Peytoia nathorsti
Schinderhannes bartelsi
Buccaspinea cooperi
Cambroraster falcatus
Hurdia triangulata
Hurdia victoria
Hurdiidae
Radiodonta
Fuxianhuia xiaoshibaensis
Chengjiangocaris kunmingensis
Misszhouia longicaudata
Kuamaia lata
upper stem group Euarthropoda
Triops cancriformis
Leanchoilia superlata
Alalcomenaeus sp
Limulus polyphemus
crown group Euarthropoda
Deuteropoda
0.2
b
Scalidophora
crown group Tardigrada
Panarthropoda
crown group Onychophora
Euarthropoda
gilled lobopodians
Opabiniidae
Castle Bank
Deuteropoda
upper stem group Euarthropoda
crown group Euarthropoda
Amplectobeluidae + Anomalocarididae
Radiodonta
Hurdiidae
0.2
c
Scalidophora
Panarthropoda
crown group Onychophora
crown group Tardigrada
Tactopoda
Euarthropoda
gilled lobopodians
Opabiniidae
Castle Bank
Deuteropoda
upper stem group Euarthropoda
crown group Euarthropoda
Radiodonta
2.0

Supplementary Figure 6 (previous page). Results of the phylogenetic analyses considering the two Castle Bank specimens as separate species. (a) Majority-rule consensus tree retrieved with BI maximum information strategy. (b) Majority-rule consensus tree retrieved with BI minimum assumptions strategy. (c) Strict consensus tree retrieved with Maximum Parsimony, concavity constant k = 3. Nine most parsimonious trees, 241 steps, consistency index: 0.680, retention index: 0.885. Numbers above nodes in (a, b) indicate posterior probabilities. Numbers above and below nodes in (c) indicate resampling support. Above: jackknife; below: symmetric.

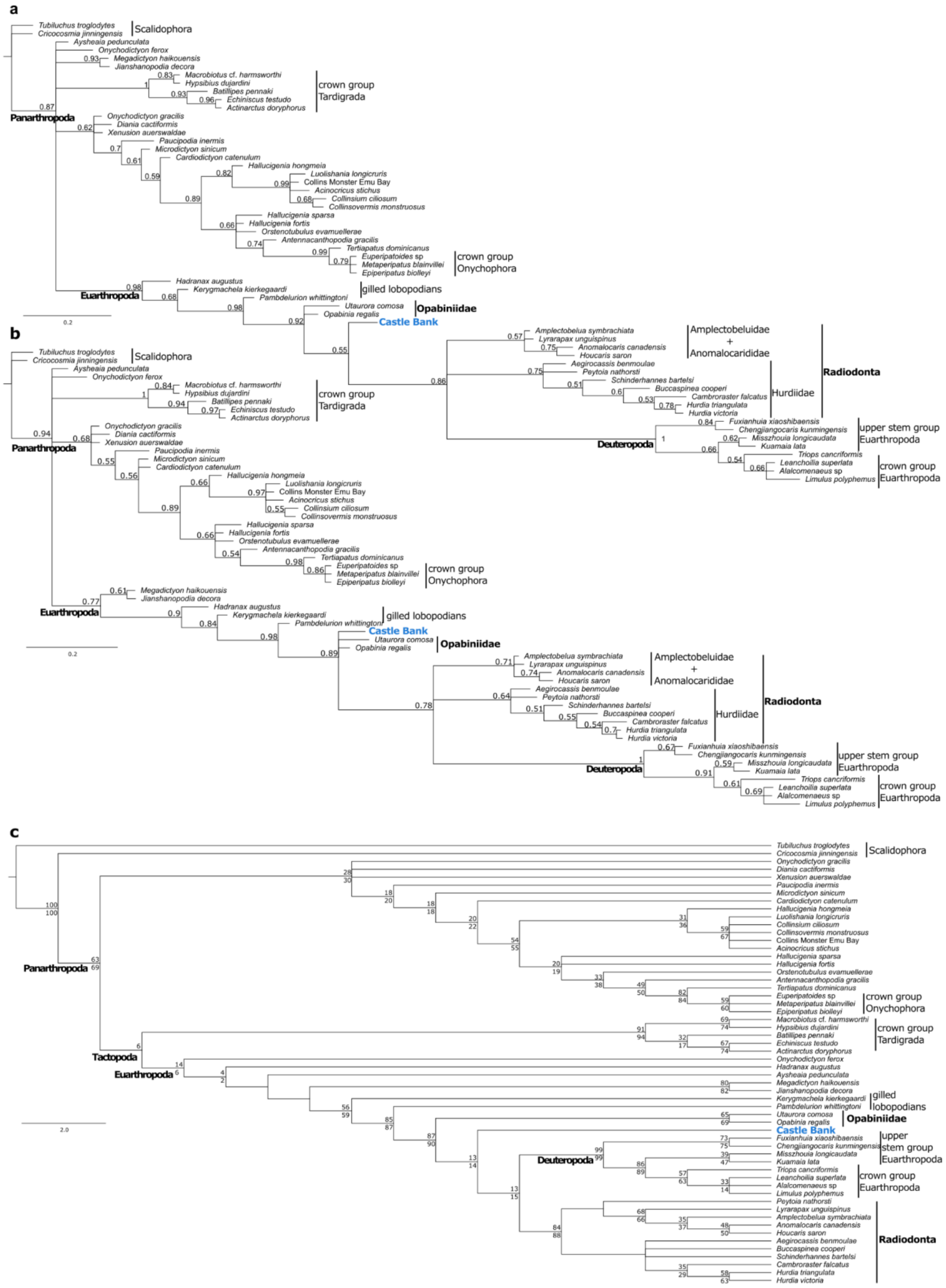
a
Tubiluchus troglodytes
Cricocosmia jinningensis
Scalidophora
Aysheaia pedunculata
Onychodictyon ferox
Megadictyon haikouensis
Jianshanopodia decora
Macrobiotus cf. harmsworthi
Hypsibius dujardini
Batillipes pennaki
Echiniscus testudo
Actinarctus doryphorus
crown group Tardigrada
Panarthropoda
Onychodictyon gracilis
Diania cactiformis
Xenusion auerswaldae
Paucipodia inermis
Microdictyon sinicum
Cardiodictyon catenulum
Hallucigenia hongmeia
Luolishania longicruris
Collins Monster Emu Bay
Acinocricus stichus
Collinsium ciliosum
Collinsovermis monstruosus
Hallucigenia sparsa
Hallucigenia fortis
Orstenotubulus evamuellerae
Antennacanthopodia gracilis
Tertiapatus dominicanus
Euperipatoides sp
Metaperipatus blainvillei
Epiperipatus biolleyi
crown group Onychophora
Euarthropoda
Hadranax augustus
Kerygmachela kierkegaardi
Pambdelurion whittingtoni
gilled lobopodians
Utaurora comosa
Opabinia regalis
Opabiniidae
Castle Bank
Amplectobelua symbrachiata
Lyrarapax unguispinus
Anomalocaris canadensis
Houcaris saron
Amplectobeluidae + Anomalocarididae
Aegirocassis benmoulae
Peytoia nathorsti
Schinderhannes bartelsi
Buccaspinea cooperi
Cambroraster falcatus
Hurdia triangulata
Hurdia victoria
Hurdiidae
Radiodonta
Deuteropoda
Fuxianhuia xiaoshibaensis
Chengjiangocaris kunmingensis
Misszhouia longicaudata
Kuamaia lata
upper stem group Euarthropoda
Triops cancriformis
Leanchoilia superlata
Alalcomenaeus sp
Limulus polyphemus
crown group Euarthropoda
0.2
b
Panarthropoda
Euarthropoda
Castle Bank
Opabiniidae
Radiodonta
Deuteropoda
0.2
c
Panarthropoda
Tactopoda
Euarthropoda
Deuteropoda
Castle Bank
Opabiniidae
gilled lobopodians
upper stem group Euarthropoda
crown group Euarthropoda
crown group Onychophora
crown group Tardigrada
Radiodonta
Scalidophora
2.0

Supplementary Figure 7 (previous page). Results of the phylogenetic analyses considering the two Castle Bank specimens as one terminal. (a) Majority-rule consensus tree retrieved with BI maximum information strategy. (b) Majority-rule consensus tree retrieved with BI minimum assumptions strategy. (c) Strict consensus tree retrieved with Maximum Parsimony, concavity constant k = 3. Nine most parsimonious trees, 240 steps, consistency index: 0.685, retention index: 0.887. Numbers above nodes in (a, b) indicate posterior probabilities. Numbers above and below nodes in (c) indicate resampling support. Above: jackknife; below: symmetric.

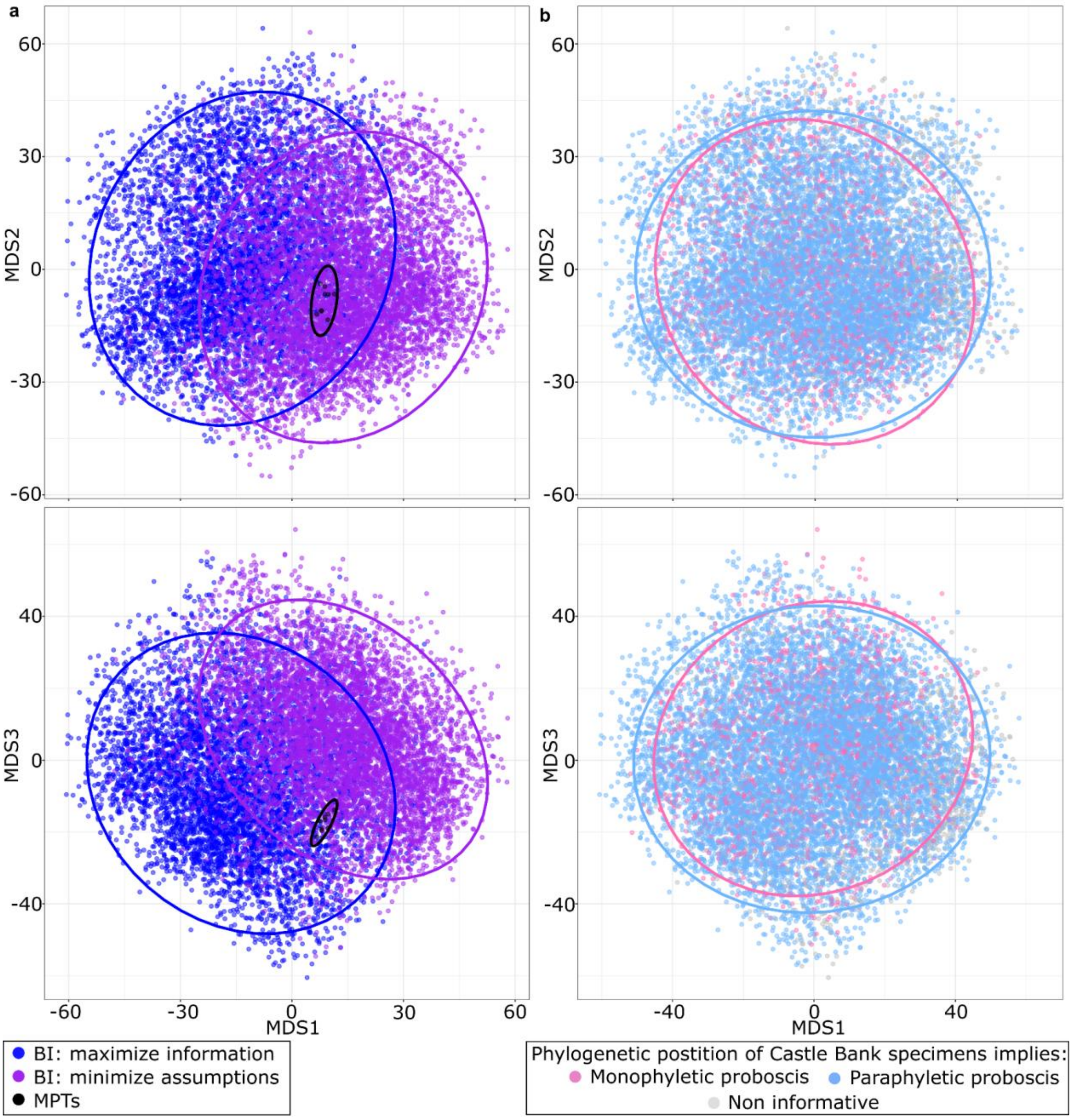

Supplementary Figure 8. Treespace visualization of phylogenetic analyses treating Castle Bank specimens as two distinct species. (a) Trees coloured by phylogenetic method. (b) Trees coloured by whether position of Castle Bank specimens implies a monophyletic group of proboscis-bearing stem group euarthropods, or whether instead it implies a paraphyletic grade of proboscis-bearing stem group euarthropods. Trees labelled as non informative did not recover any of the topologies in **Supplementary Table 1**. Abbreviations: BI, Bayesian Inference; MPTs, most parsimonious trees. Ellipses capture the 95% confidence level for the data, assuming a multivariate t-distribution.

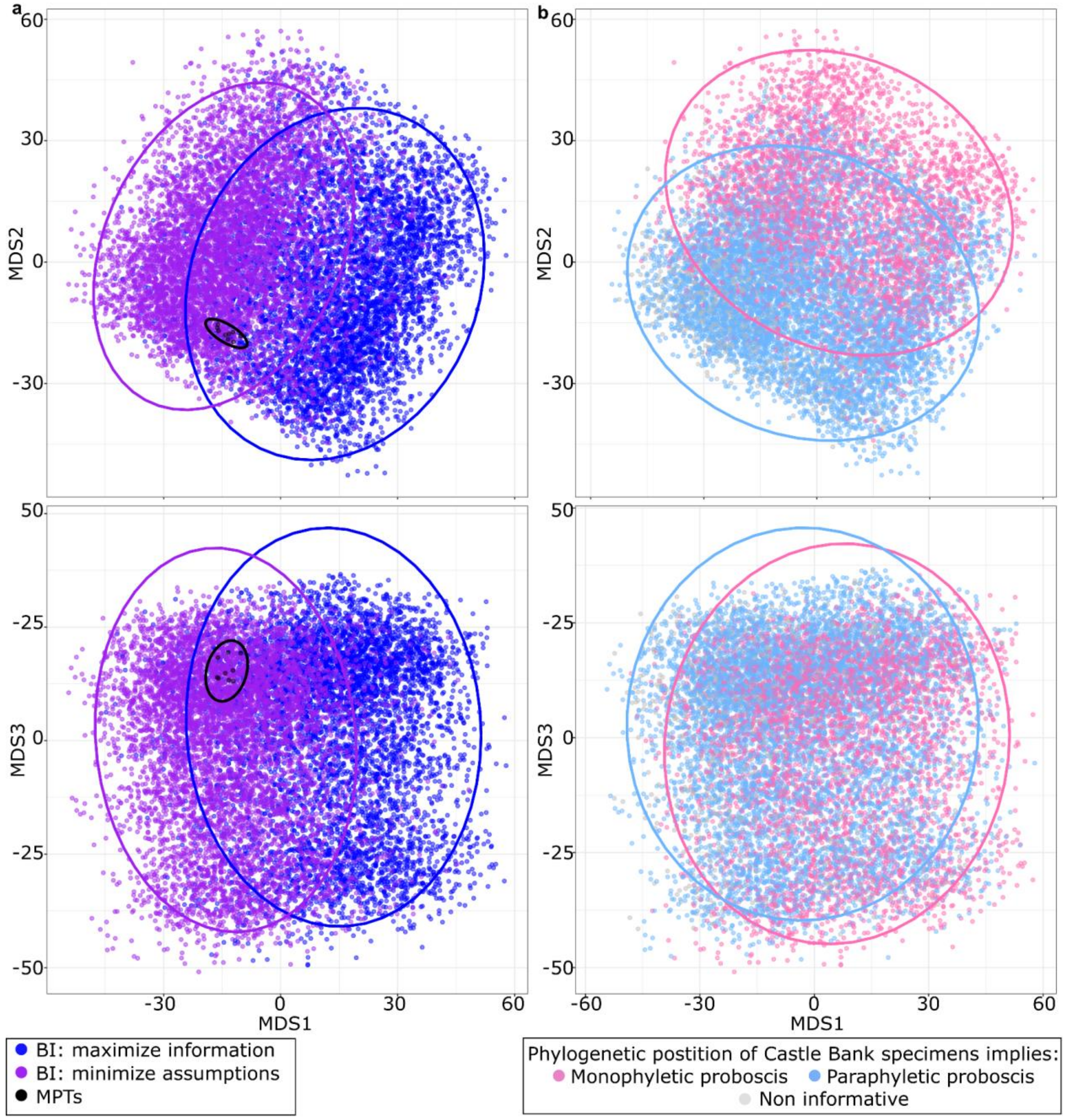

Supplementary Figure 9. Treespace visualization of phylogenetic analyses treating Castle Bank specimens as a single terminal. (a) Trees coloured by phylogenetic method. (b) Trees coloured by whether position of Castle Bank specimens implies a monophyletic group of proboscis-bearing stem group euarthropods, or whether instead it implies a paraphyletic grade of proboscis-bearing stem group euarthropods. Trees labelled as non informative did not recover any of the topologies in **Supplementary Table 2**. Abbreviations: BI, Bayesian Inference; MPTs, most parsimonious trees. Ellipses capture the 95% confidence level for the data, assuming a multivariate t-distribution.

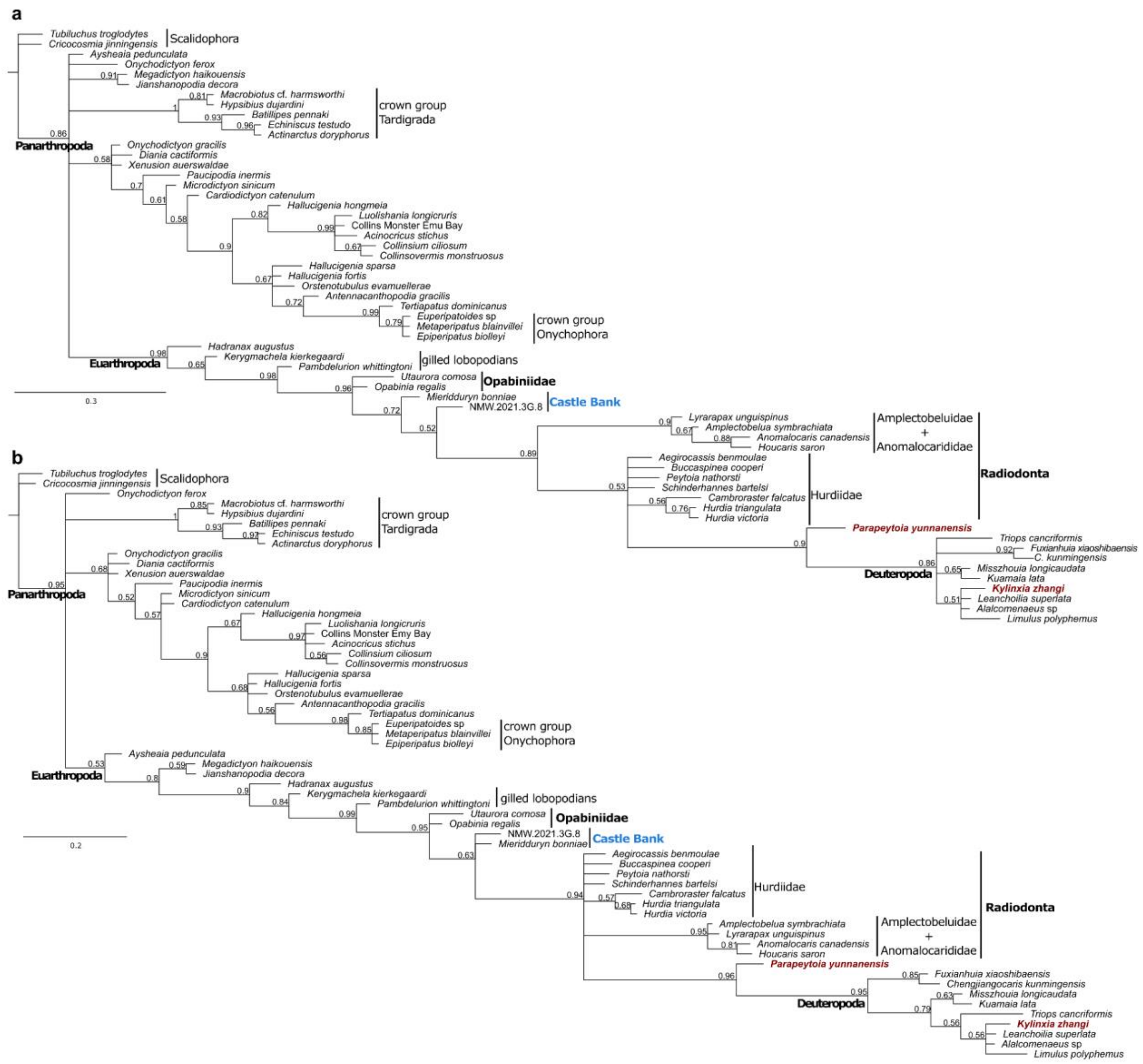

Supplementary Figure 10. Results of phylogenetic analyses including *Kylinxia* and *Parapeytoia*, with *Parapeytoia* anteriormost appendages interpreted as protocerebral, following Budd (2021). Numbers above nodes indicate posterior probabilities. (a) Majority rule consensus tree retrieved with BI maximum information strategy. (b) Majority rule consensus tree retrieved with BI minimum assumptions strategy.

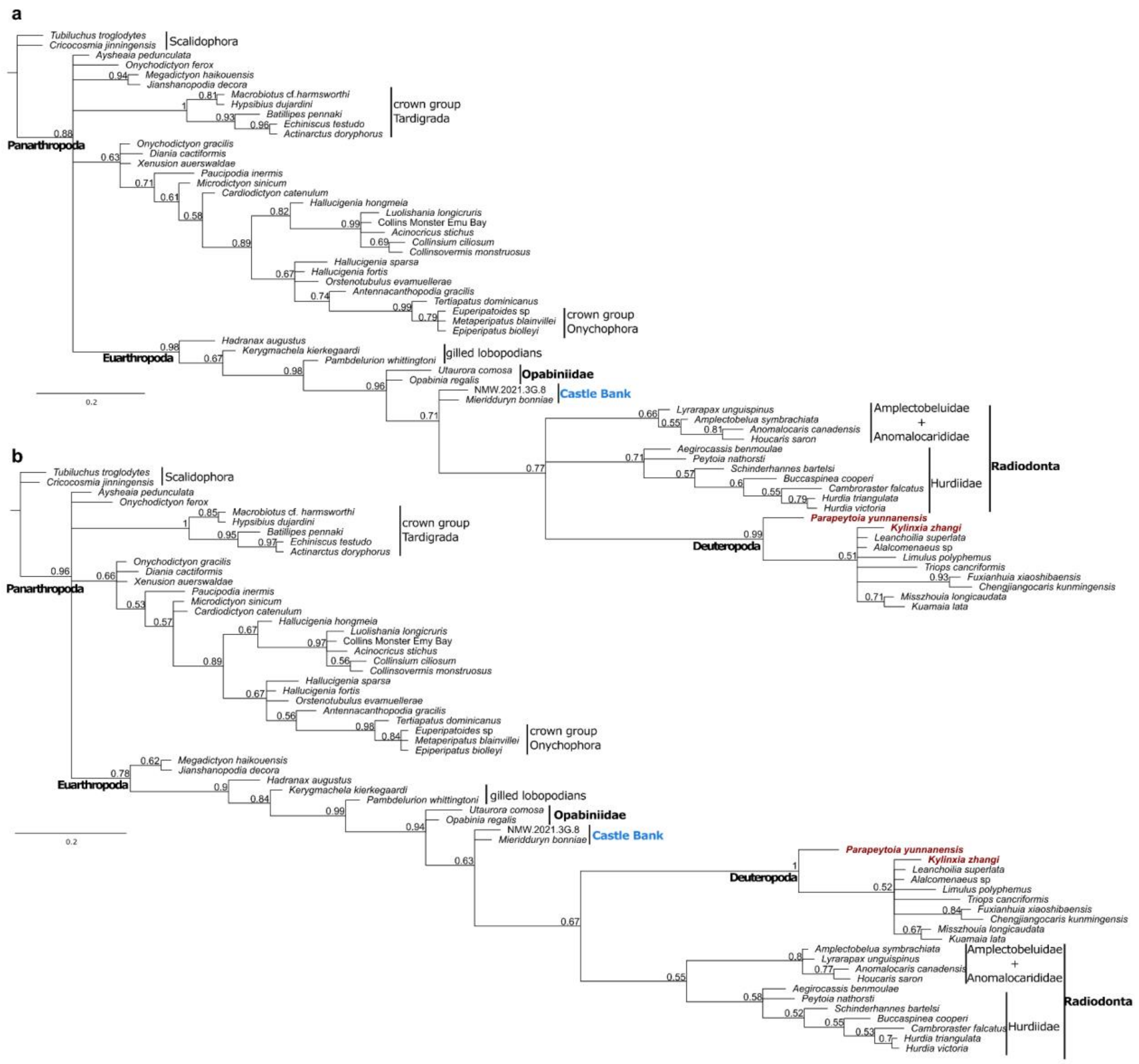

Supplementary Figure 11. Results of phylogenetic analyses including *Kylinxia* and *Parapeytoia*, with *Parapeytoia* anteriormost appendages interpreted as deutocerebral. Numbers above nodes indicate posterior probabilities. (a) Majority rule consensus tree retrieved with BI maximum information strategy. (b) Majority rule consensus tree retrieved with BI minimum assumptions strategy.

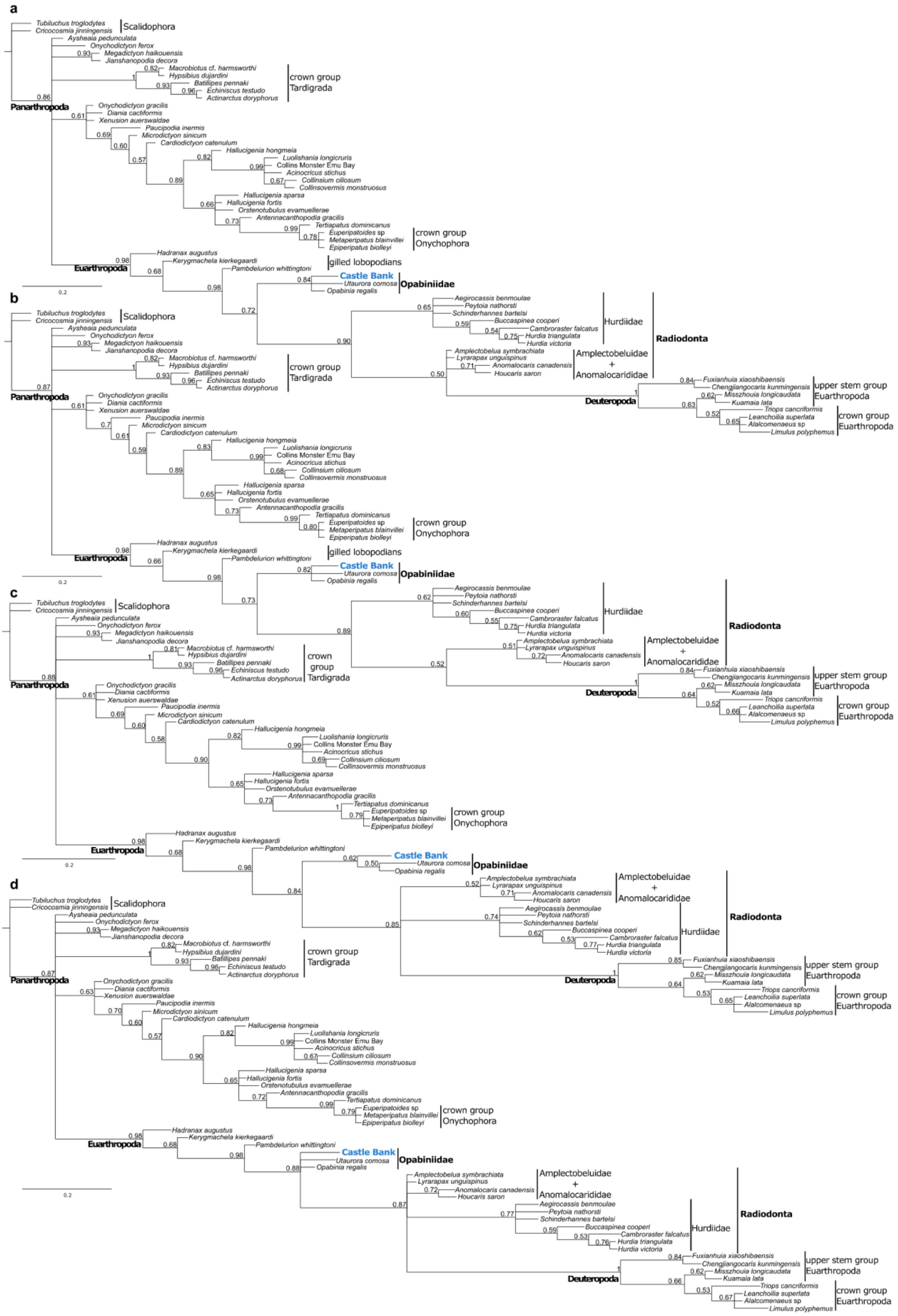
a
b
c
d
Tubiluchus troglodytes
Cricocosmia jinningensis
Scalidophora
Aysheaia pedunculata
Onychodictyon ferox
Megadictyon haikouensis
Jianshanopodia decora
Macrobiotus cf. harmsworthi
Hypsibius dujardini
Batillipes pennaki
Echiniscus testudo
Actinarctus doryphorus
crown group Tardigrada
Panarthropoda
Onychodictyon gracilis
Diania cactiformis
Xenusion auerswaldae
Paucipodia inermis
Microdictyon sinicum
Cardiodictyon catenulum
Hallucigenia hongmeia
Luolishania longicruris
Collins Monster Emu Bay
Acinocricus stichus
Collinsium ciliosum
Collinsovermis monstruosus
Hallucigenia sparsa
Hallucigenia fortis
Orstenotubulus evamuellerae
Antennacanthopodia gracilis
Tertiapatus dominicanus
Euperipatoides sp
Metaperipatus blainvillei
Epiperipatus biolleyi
crown group Onychophora
Hadranax augustus
Kerygmachela kierkegaardi
gilled lobopodians
Euarthropoda
Pambdelurion whittingtoni
Castle Bank
Utaurora comosa
Opabinia regalis
Opabiniidae
Aegirocassis benmoulae
Peytoia nathorsti
Schinderhannes bartelsi
Buccaspinea cooperi
Cambroraster falcatus
Hurdia triangulata
Hurdia victoria
Hurdiidae
Radiodonta
Amplectobelua symbrachiata
Lyrarapax unguispinus
Anomalocaris canadensis
Houcaris saron
Amplectobeluidae + Anomalocarididae
Deuteropoda
Fuxianhuia xiaoshibaensis
Chengjiangocaris kunmingensis
Misszhouia longicaudata
Kuamaia lata
upper stem group Euarthropoda
Triops cancriformis
Leanchoilia superlata
Alalcomenaeus sp
Limulus polyphemus
crown group Euarthropoda
0.2

Supplementary Figure 12 (previous page). Results of phylogenetic analyses treating some morphological features shared between Castle Bank specimens and radiodonts as convergent, treating Castle Bank specimens as a single terminal. Numbers above nodes indicate posterior probabilities. Morphological features considered: dorsal carapace, dorsal spines on protocerebral appendage, and strengthening rays in swimming flaps. (a) All characters considered convergent (coding for 13 characters changed, 2 new characters introduced). (b) Carapace characters considered convergent (coding for 11 characters changed, 1 new character introduced). (c) Dorsal spines considered convergent (coding for 1 character changed, 1 new character introduced). (d) Strengthening rays considered convergent (coding for 1 character changed).

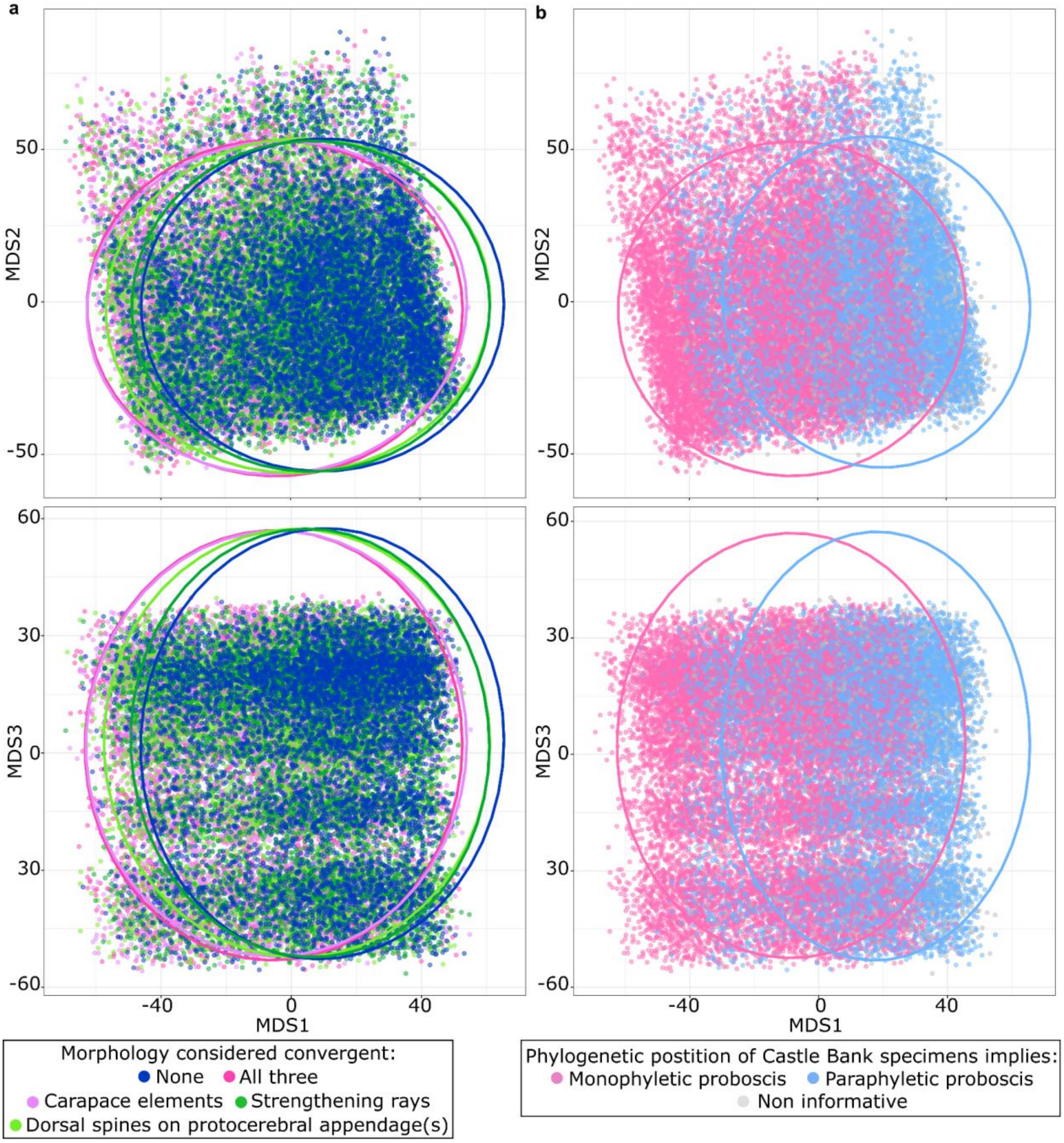

Supplementary Figure 13. Treespace visualization of phylogenetic analyses treating some morphological features shared between Castle Bank specimens and radiodonts as convergent, treating Castle Bank specimens as a single terminal. All analyses run using the 'maximum information' strategy. (a) Trees coloured by morphological feature considered convergent. (b) Trees coloured by whether position of Castle Bank specimens implies a monophyletic group of proboscis-bearing stem group euarthropods, or whether instead it implies a paraphyletic grade of proboscis-bearing stem group euarthropods. Trees labelled as non informative did not recover any of the topologies in **Supplementary Table 3**. Full results for these analyses figured in **Supplementary Figure 12** and presented in **Supplementary Table 3.** Ellipses capture the 95% confidence level for the data, assuming a multivariate t-distribution.

Supplementary References

1. Whittington, H. B. The enigmatic animal *Opabinia regalis*, middle Cambrian, Burgess Shale, British Columbia. *Philos. Trans. R. Soc. London. B, Biol. Sci.* **271**, 1–43 (1975).
2. Pates, S., Wolfe, J. M., Lerosey-Aubril, R., Daley, A. C. & Ortega-Hernández, J. New opabiniid diversifies the weirdest wonders of the euarthropod stem group. *Proc. R. Soc. B* **289**, 20212093 (2022).
3. Daley, A. C. & Budd, G. E. New anomalocaridid appendages from the Burgess Shale, Canada. *Palaeontology* **53**, 721–738 (2010).
4. Daley, A. C. & Edgecombe, G. D. Morphology of *Anomalocaris canadensis* from the Burgess Shale. *J. Paleontol.* **88**, 68–91 (2014).
5. Guo, J. *et al.* A new radiodont (stem Euarthropoda) frontal appendage with a mosaic of characters from the Cambrian (Series 2 Stage 3) Chengjiang biota. *Pap. Palaeontol.* **5**, 99–110 (2019).
6. Cong, P., Daley, A. C., Edgecombe, G. D. & Hou, X. The functional head of the Cambrian radiodontan (stem-group Euarthropoda) *Amplectobelua symbrachiata*. *BMC Evol. Biol.* **17**, 1–23 (2017).
7. Daley, A. C., Budd, G. E., Caron, J.-B., Edgecombe, G. D. & Collins, D. The Burgess Shale Anomalocaridid *Hurdia* and Its Significance for Early Euarthropod Evolution. *Science.* **323**, 1597–1600 (2009).
8. Moysiuk, J. & Caron, J. B. A new hurdiid radiodont from the Burgess Shale evinces the exploitation of Cambrian infaunal food sources. *Proc. R. Soc. B Biol. Sci.* **286**, (2019).
9. Van Roy, P., Daley, A. C. & Briggs, D. E. G. Anomalocaridid trunk limb homology revealed by a giant filter-feeder with paired flaps. *Nature* **522**, 77–80 (2015).
10. Liu, J. *et al.* Origin of raptorial feeding in juvenile euarthropods revealed by a Cambrian radiodontan. *Natl. Sci. Rev.* **5**, 863–869 (2018).
11. Zeng, H., Zhao, F., Yin, Z. & Zhu, M. Morphology of diverse radiodontan head sclerites from the early Cambrian Chengjiang Lagerstätte, south-west China. *J. Syst. Palaeontol.* **16**, 1–37 (2018).
12. Ortega-Hernández, J. Homology of Head Sclerites in Burgess Shale Euarthropods. *Curr. Biol.* **25**, 1625–1631 (2015).
13. Vinther, J., Porras, L., Young, F. J., Budd, G. E. & Edgecombe, G. D. The mouth apparatus of the Cambrian gilled lobopodian *Pambdelurion whittingtoni*. *Palaeontology* **59**, 841–849 (2016).
14. Daley, A. C. & Bergström, J. The oral cone of *Anomalocaris* is not a classic '*Peytoia*'. *Naturwissenschaften* **99**, 501–504 (2012).
15. Cong, P. *et al.* New radiodonts with gnathobase-like structures from the Cambrian Chengjiang biota and implications for the systematics of Radiodonta. *Pap. Palaeontol.* **4**, 605–621 (2018).
16. Lerosey-Aubril, R. & Pates, S. New suspension-feeding radiodont suggests evolution of microplanktivory in Cambrian macronekton. *Nat. Commun.* **9**, 1–9 (2018).
17. Daley, A. C., Budd, G. E. & Caron, J.-B. Morphology and systematics of the anomalocaridid arthropod *Hurdia* from the Middle Cambrian of British Columbia and Utah. *J. Syst. Palaeontol.* **11**, 743–787 (2013).

18. Budd, G. E. & Daley, A. C. The lobes and lobopods of *Opabinia regalis* from the middle Cambrian Burgess Shale. *Lethaia* **45**, 83–95 (2012).

19. Budd, G. A Cambrian gilled lobopod from Greenland. *Nature* **364**, 709–711 (1993).

20. Young, F. J. & Vinther, J. Onychophoran-like myoanatomy of the Cambrian gilled lobopodian *Pambdelurion whittingtoni*. *Palaeontology* **60**, 27–54 (2017).

21. Ma, X., Edgecombe, G. D., Legg, D. A. & Hou, X. The morphology and phylogenetic position of the Cambrian lobopodian *Diania cactiformis*. *J. Syst. Palaeontol.* **12**, 445–457 (2014).

22. Liu, J. & Dunlop, J. A. Cambrian lobopodians: A review of recent progress in our understanding of their morphology and evolution. *Palaeogeogr. Palaeoclimatol. Palaeoecol.* **398**, 4–15 (2014).

23. Pates, S. *et al.* The diverse radiodont fauna from the Marjum Formation of Utah, USA (Cambrian: Drumian). *PeerJ* **9**, 1–43 (2021).

24. Budd, G. E. Stem group arthropods from the Lower Cambrian Sirius Passet fauna of North Greenland. in *Arthropod Relationships* 125–138 (Springer Netherlands, 1998). doi:10.1007/978-94-011-4904-4_11.

25. Vannier, J. *et al. Tuzoia*: Morphology and lifestyle of a large bivalved Arthropod of the Cambrian seas. *J. Paleontol.* **81**, 445–471 (2007).

26. Williams, M., Siveter, D. J. & Peel, J. S. *Isoxys* (Arthropoda) from the Early Cambrian Sirius Passet Lagerstätte, North Greenland. *J. Paleontol.* **70**, 947–954 (1996).

27. Legg, D. A., Sutton, M. D., Edgecombe, G. D. & Caron, J. B. Cambrian bivalved arthropod reveals origin of arthrodization. *Proc. R. Soc. B Biol. Sci.* **279**, 4699–4704 (2012).

28. Budd, G. E. The origin and evolution of the euarthropod labrum. *Arthropod Struct. Dev.* **62**, 101048 (2021).

29. Chen, J. Y., Ramsköld, L. & Zhou, G. Q. Evidence for Monophyly and Arthropod Affinity of Cambrian Giant Predators. *Science .* **264**, 1304–1308 (1994).

30. Wright, A. M. & Hillis, D. M. Bayesian Analysis Using a Simple Likelihood Model Outperforms Parsimony for Estimation of Phylogeny from Discrete Morphological Data. *PLoS One* **9**, e109210 (2014).

31. Smith, M. R. Bayesian and parsimony approaches reconstruct informative trees from simulated morphological datasets. *Biol. Lett.* **15**, (2019).

32. Wolfe, J. M. & Hegna, T. A. Testing the phylogenetic position of Cambrian pancrustacean larval fossils by coding ontogenetic stages. *Cladistics* **30**, 366–390 (2014).

33. Bapst, D. W., Schreiber, H. A. & Carlson, S. J. Combined Analysis of Extant Rhynchonellida (Brachiopoda) using Morphological and Molecular Data. *Syst. Biol.* **67**, 32–48 (2018).

34. Wright, A. M., Lloyd, G. T. & Hillis, D. M. Modeling character change heterogeneity in phylogenetic analyses of morphology through the use of priors. *Syst. Biol.* **65**, 602–611 (2016).

35. Zeng, H., Zhao, F., Niu, K., Zhu, M. & Huang, D. An early Cambrian euarthropod with radiodont-like raptorial appendages. *Nature* **588**, 101–105 (2020).

36. Tanaka, G., Hou, X., Ma, X., Edgecombe, G. D. & Strausfeld, N. J. Chelicerate neural ground pattern in a Cambrian great appendage arthropod. *Nature* **502**, 364–367 (2013).

37. Ortega-Hernández, J., Lerosey-Aubril, R. & Pates, S. Proclivity of nervous system preservation in Cambrian Burgess Shale-type deposits. *Proc. R. Soc. B Biol. Sci.* **286**, 20192370 (2019).

38. Liu, Y., Ortega-Hernández, J., Zhai, D. & Hou, X. A Reduced Labrum in a Cambrian Great-Appendage Euarthropod. *Curr. Biol.* **30**, 3057-3061.e2 (2020).

39. Hou, X., Bergström, J. & Ahlberg, P. *Anomalocaris* and other large animals in the Lower Cambrian Chengjiang fauna of southwest China. *GFF* **117**, 163–183 (1995).

40. Haug, J. T., Briggs, D. E. G. & Haug, C. Morphology and function in the Cambrian Burgess Shale megacheiran arthropod *Leanchoilia superlata* and the application of a descriptive matrix. *BMC Evol. Biol.* **12**, (2012).

41. Aria, C., Zhao, F., Zeng, H., Guo, J. & Zhu, M. Fossils from South China redefine the ancestral euarthropod body plan. *BMC Evol. Biol.* **20**, (2020).

42. Cong, P., Ma, X., Hou, X., Edgecombe, G. D. & Strausfeld, N. J. Brain structure resolves the segmental affinity of anomalocaridid appendages. *Nature* **513**, 538–542 (2014).

43. Zhang, C. *et al.* Differentiated appendages in *Isoxys* illuminate origin of arthropodization. *Res. Sq. Prepr.* 1–17 (2021) doi:10.21203/rs.3.rs-861892/v1.

44. Budd, G. E. On the origin and evolution of major morphological characters. *Biol. Rev.* **81**, 609–628 (2007).

45. Ortega-Hernández, J. Making sense of 'lower' and 'upper' stem-group Euarthropoda, with comments on the strict use of the name Arthropoda von Siebold, 1848. *Biol. Rev.* **91**, 255–273 (2016).

46. Fu, D. *et al.* The evolution of biramous appendages revealed by a carapace-bearing Cambrian arthropod. *Philos. Trans. R. Soc. B* **377**, (2022).

47. Ortega-Hernández, J., Janssen, R. & Budd, G. E. Origin and evolution of the panarthropod head – A palaeobiological and developmental perspective. *Arthropod Struct. Dev.* **46**, 354–379 (2017).

48. Yang, J., Ortega-Hernández, J., Lan, T., Hou, J. B. & Zhang, X. G. A predatory bivalved euarthropod from the Cambrian (Stage 3) Xiaoshiba Lagerstätte, South China. *Sci. Rep.* **6**, (2016).

49. Lamsdell, J. C., Stein, M. & Selden, P. A. *Kodymirus* and the case for convergence of raptorial appendages in Cambrian arthropods. *Naturwissenschaften* **100**, 811–825 (2013).

50. Jockusch, E. L. Developmental and evolutionary perspectives on the origin and diversification of arthropod appendages. *Integr. Comp. Biol.* **57**, 533–545 (2017).

51. Pates, S. & Daley, A. C. The Kinzers Formation (Pennsylvania, USA): The most diverse assemblage of Cambrian Stage 4 radiodonts. *Geol. Mag.* **156**, 1233–1246 (2019).

52. Pates, S., Daley, A. C., Edgecombe, G. D., Cong, P. & Lieberman, B. S. Systematics, preservation and biogeography of radiodonts from the southern Great Basin, USA, during the upper Dyeran (Cambrian Series 2, Stage 4). *Pap. Palaeontol.* **7**, 235–262 (2021).

53. Jiao, D.-G. *et al.* The endemic radiodionts of the Cambrian Stage 4 Guanshan Biota of South China. *Acta Palaeontol. Pol.* **66**, 255–274 (2021).
