# Supplementary material for: Ordovician opabiniid-like animals and the role of the proboscis in euarthropod head evolution
Source: Nat Commun. 2022 Nov 15;13:6969. doi: 10.1038/s41467-022-34204-w (PMC9666559; doi:10.1038/s41467-022-34204-w)
Supplement: Supplementary file 3 — Reporting Summary [file 41467_2022_34204_MOESM3_ESM.pdf]

nature portfolio

Corresponding author(s): Stephen Pates
Last updated by author(s): Jun 17, 2022

Reporting Summary

Nature Portfolio wishes to improve the reproducibility of the work that we publish. This form provides structure for consistency and transparency in reporting. For further information on Nature Portfolio policies, see our Editorial Policies and the Editorial Policy Checklist.

Statistics

For all statistical analyses, confirm that the following items are present in the figure legend, table legend, main text, or Methods section.

n/a	Confirmed	
☒	☐	The exact sample size (*n*) for each experimental group/condition, given as a discrete number and unit of measurement
☒	☐	A statement on whether measurements were taken from distinct samples or whether the same sample was measured repeatedly
☒	☐	The statistical test(s) used AND whether they are one- or two-sided<br>*Only common tests should be described solely by name; describe more complex techniques in the Methods section.*
☒	☐	A description of all covariates tested
☒	☐	A description of any assumptions or corrections, such as tests of normality and adjustment for multiple comparisons
☐	☒	A full description of the statistical parameters including central tendency (e.g. means) or other basic estimates (e.g. regression coefficient) AND variation (e.g. standard deviation) or associated estimates of uncertainty (e.g. confidence intervals)
☒	☐	For null hypothesis testing, the test statistic (e.g. *F*, *t*, *r*) with confidence intervals, effect sizes, degrees of freedom and *P* value noted<br>*Give P values as exact values whenever suitable.*
☐	☒	For Bayesian analysis, information on the choice of priors and Markov chain Monte Carlo settings
☒	☐	For hierarchical and complex designs, identification of the appropriate level for tests and full reporting of outcomes
☒	☐	Estimates of effect sizes (e.g. Cohen's *d*, Pearson's *r*), indicating how they were calculated

Our web collection on statistics for biologists contains articles on many of the points above.

Software and code

Policy information about availability of computer code

Data collection	Inkscape, Glimpse, R, TNT and MrBayes are all open source
Data analysis	R code, nexus files, and TNT code provided in OSF project, link provided in Data Availability part of manuscript

For manuscripts utilizing custom algorithms or software that are central to the research but not yet described in published literature, software must be made available to editors and reviewers. We strongly encourage code deposition in a community repository (e.g. GitHub). See the Nature Portfolio guidelines for submitting code & software for further information.

Data

Policy information about availability of data

All manuscripts must include a data availability statement. This statement should provide the following information, where applicable:

- Accession codes, unique identifiers, or web links for publicly available datasets
- A description of any restrictions on data availability
- For clinical datasets or third party data, please ensure that the statement adheres to our policy

Supplementary data files are available at MorphoBank (www.morphobank.org, doi:10.7934/P4146) and in the Open Science Framework (doi: 10.17605/OSF.IO/4FTZY). Specimens are accessioned at Amgueddfa Cymru—National Museum Wales, Cardiff, UK. Nomenclatural acts relating to the new taxon have been registered with ZooBank, LSID urn:lsid:zoobank.org:pub:6216E87D-6FC9-4A32-B5CF-5E8EF3D13440 (publication), LSID urn:lsid:zoobank.org:act:9860A52F-4B3F-4B6F-AE96-CC090DB51046 (genus), LSID urn:lsid:zoobank.org:act:9F00C780-C781-4EDB-9C9C-52BA92F92171 .

Human research participants

Policy information about studies involving human research participants and Sex and Gender in Research.

Reporting on sex and gender	na
Population characteristics	na
Recruitment	na
Ethics oversight	na

Note that full information on the approval of the study protocol must also be provided in the manuscript.

Field-specific reporting

Please select the one below that is the best fit for your research. If you are not sure, read the appropriate sections before making your selection.

☐ Life sciences ☐ Behavioural & social sciences ☒ Ecological, evolutionary & environmental sciences

For a reference copy of the document with all sections, see nature.com/documents/nr-reporting-summary-flat.pdf

Ecological, evolutionary & environmental sciences study design

All studies must disclose on these points even when the disclosure is negative.

Study description	A fossil study. Describing a new taxon and a possible second from two specimens. Placing this into an existing morphological dataset which was then updated, and phylogenetic analyses run.
Research sample	Additional material for this study comes from two fossil specimens, all the material that is currently available.
Sampling strategy	All fragments of rock that possibly contained fossil material were studied.
Data collection	Fossils collected in the field over months by JPB and LAM
Timing and spatial scale	Fossils were collected continuously on days with suitable weather.
Data exclusions	No data were excluded
Reproducibility	na
Randomization	na
Blinding	na

Did the study involve field work? ☒ Yes ☐ No

Field work, collection and transport

Field conditions	Specimens were collected from a small (10m-wide) quarry on private land in Wales, and collections have been made over two years of excavations. Collecting is limited to dry conditions with good light (ideally sunny), since the small fossils are otherwise very difficult to see, and specimens are likely to be missed or damaged accidentally.
Location	The location is a small, privately owned domestic farm quarry near Llandrindod, Wales, UK. Exact co-ordinates are provided with the specimens and are available to researchers (with the specimens at the National Museum Wales), but are not being published in order to protect the site.
Access & import/export	The quarry is located in a livestock (sheep grazing) field adjacent to the owners' house. Full permission has been granted by the landowners for excavation and deposition of specimens, and the land does not fall under any restrictions requiring permits for the work. Similarly, the specimens are retained in the country of origin (UK), deposited in the National Museum Wales; however, in any case, no permits would have been required for export of this material from the UK.
Disturbance	The excavation work is small-scale and painstaking, and the potential for disturbance in a field used only for sheep grazing is minimal; no birds nest nearby, and the short grazed grass is of minimal significance for biodiversity. No powered tools were used, except for

one use of a tractor to extend one part of the quarry; this was done by the farmer who owns the sheep, who can be assumed to have acted appropriately. We do not play music or cause other noise pollution, beyond the sound of gentle hammer use. The sheep are not generally disturbed by quiet hammering, as they usually come to watch; if they need to move away, the field extends for another 200m, and they are free to do so. Disturbance to invertebrates in the quarry was mitigated by carefully moving any beetles (generally carabids), centipedes, worms, lygaeid bugs, spiders or other creatures that were considered to be in danger of being harmed by rocks.

Reporting for specific materials, systems and methods

We require information from authors about some types of materials, experimental systems and methods used in many studies. Here, indicate whether each material, system or method listed is relevant to your study. If you are not sure if a list item applies to your research, read the appropriate section before selecting a response.

Materials & experimental systems

n/a	Involved in the study
☒	☐ Antibodies
☒	☐ Eukaryotic cell lines
☐	☒ Palaeontology and archaeology
☒	☐ Animals and other organisms
☐	☒ Clinical data
☒	☐ Dual use research of concern

Methods

n/a	Involved in the study
☒	☐ ChIP-seq
☒	☐ Flow cytometry
☒	☐ MRI-based neuroimaging

Palaeontology and Archaeology

Specimen provenance	Private land near Llandrindod Wells, Wales, UK. Permission of the landowner was obtained prior to fieldwork and fossil collection.
Specimen deposition	Amgueddfa Cymru - National Museum Wales
Dating methods	No new dates.

☒ Tick this box to confirm that the raw and calibrated dates are available in the paper or in Supplementary Information.

Ethics oversight	No ethical oversight is required as fossils were from private land in the UK collected by people who live in the area.

Note that full information on the approval of the study protocol must also be provided in the manuscript.

Clinical data

Policy information about clinical studies

All manuscripts should comply with the ICMJEguidelines for publication of clinical research and a completedCONSORT checklist must be included with all submissions.

Clinical trial registration	*Provide the trial registration number from ClinicalTrials.gov or an equivalent agency.*
Study protocol	*Note where the full trial protocol can be accessed OR if not available, explain why.*
Data collection	*Describe the settings and locales of data collection, noting the time periods of recruitment and data collection.*
Outcomes	*Describe how you pre-defined primary and secondary outcome measures and how you assessed these measures.*
